# Targeting of lipid metabolism with a metabolic inhibitor cocktail eradicates peritoneal metastases in ovarian cancer cells

**DOI:** 10.1038/s42003-019-0508-1

**Published:** 2019-07-31

**Authors:** Rain R. Chen, Mingo M. H. Yung, Yang Xuan, Shijie Zhan, Leanne L. Leung, Rachel R. Liang, Thomas H. Y. Leung, Huijuan Yang, Dakang Xu, Rakesh Sharma, Karen K. L. Chan, Siew-Fei Ngu, Hextan Y. S. Ngan, David W. Chan

**Affiliations:** 1 The University of Hong Kong Shenzhen Institute of Research and Innovation (HKU-SIRI), Shenzhen, P. R. China; 2 Department of Obstetrics & Gynaecology, LKS Faculty of Medicine, The University of Hong Kong, Hong Kong SAR, P. R. China; 3 Department of Gynecological Oncology, Fudan University Shanghai Cancer Center, Fudan University, Shanghai, 200032 P.R. China; 4 Faculty of Medical Laboratory Science, Ruijin Hospital, School of Medicine, Shanghai Jiao Tong University, Shanghai, 200030 P.R. China; 5 Proteomics & Metabolomics Core Facility, LKS Faculty of Medicine, The University of Hong Kong, Hong Kong SAR, P. R. China

**Keywords:** Cancer metabolism, Cancer metabolism

## Abstract

Ovarian cancer is an intra-abdominal tumor in which the presence of ascites facilitates metastatic dissemination, and associated with poor prognosis. However, the significance of metabolic alterations in ovarian cancer cells in the ascites microenvironment remains unclear. Here we show ovarian cancer cells exhibited increased aggressiveness in ascites microenvironment via reprogramming of lipid metabolism. High lipid metabolic activities are found in ovarian cancer cells when cultured in the ascites microenvironment, indicating a metabolic shift from aerobic glycolysis to β-oxidation and lipogenesis. The reduced AMP-activated protein kinase (AMPK) activity due to the feedback effect of high energy production led to the activation of its downstream signaling, which in turn, enhanced the cancer growth. The combined treatment of low toxic AMPK activators, the transforming growth factor beta-activated kinase 1 (TAK1) and fatty acid synthase (FASN) inhibitors synergistically impair oncogenic augmentation of ovarian cancer. Collectively, targeting lipid metabolism signaling axis impede ovarian cancer peritoneal metastases.

## Abstract (web summary)

Rain Chen et al. show that lipid metabolism is highly active in ovarian cancer cells in an ascites microenvironment, causing increased aggressiveness. They show that combined treatment of AMPK activators and TAK1/FASN inhibitors could impair ovarian cancer peritoneal metastasis.

## Introduction

Ovarian cancer is one of the most common and deadly cancers in women worldwide. The high mortality rate of this cancer is due to its poor prognosis, and most cases are diagnosed at advanced stages accompanied by metastasis generated via transcoelomic spread, which are known as peritoneal metastases or peritoneal carcinomatosis^1^. Patients with peritoneal metastases and dissemination to distant sites usually have a poor prognosis. Despite advances in clinical management of this disease^2,3^, there is still no effective treatment for complete eradication of metastatic ovarian cancer cells, because of the diffuse nature of this cancer as well as the favorable tumor microenvironment in the peritoneal cavity^4^. Approximately 80% of women with ovarian carcinoma present with peritoneal metastases that are often associated with the production of ascites, and of note, most metastatic cancer cells prefer dissemination to adipose tissue-rich environments such as that in the peritoneum and omentum^5,6^. Accumulating evidence has revealed that malignant ascites and omental microenvironments provide an abundance of soluble growth factors, pro-inflammatory cytokines and fatty acids that are conducive to metastatic ovarian cancer development and invasion^7,8^. Therefore, understanding the molecular mechanisms associated with effects of the ascites or omental microenvironment on ovarian cancer cell growth and aggressiveness may enable the development of better therapeutic strategies to improve the quality of life of ovarian cancer patients. Recent evidence has shown that omental adipocytes provide fatty acids for rapid tumor growth, and also promote homing, migration, and invasion of ovarian cancer cells^9,10^. These findings suggest that metastatic ovarian cancer cells may use metabolic reprogramming to utilize lipid metabolism for tumor progression in the fatty acid-enriched microenvironment of the peritoneal cavity. However, the effect of fatty acids on ovarian cancer cell metabolism, and the mechanism by which ovarian cancer cells regulate their metabolism to facilitate tumor dissemination and invasion, remain obscure.

In this study, we report that ovarian cancer cells undergo metabolic reprogramming when cultured in omental conditioned medium (OCM), with lipid metabolism providing energy to ovarian cancer cells through AMPK/ACC signaling. On the other hand, a gradual reduction of AMPK activation due to the subsequent high ATP production led to the activation of mTOR and TAK1/NF-κB signaling, which in turn, enhanced ovarian cancer metastasis and aggressiveness. Thus, targeting lipid metabolism and/or suppressing TAK1/NF-κB signaling may be effective therapeutic strategies to prevent and treat peritoneal metastases in ovarian cancer.

## Results

### OCM enhances ovarian cancer cell oncogenic properties

**Fig. 1:**
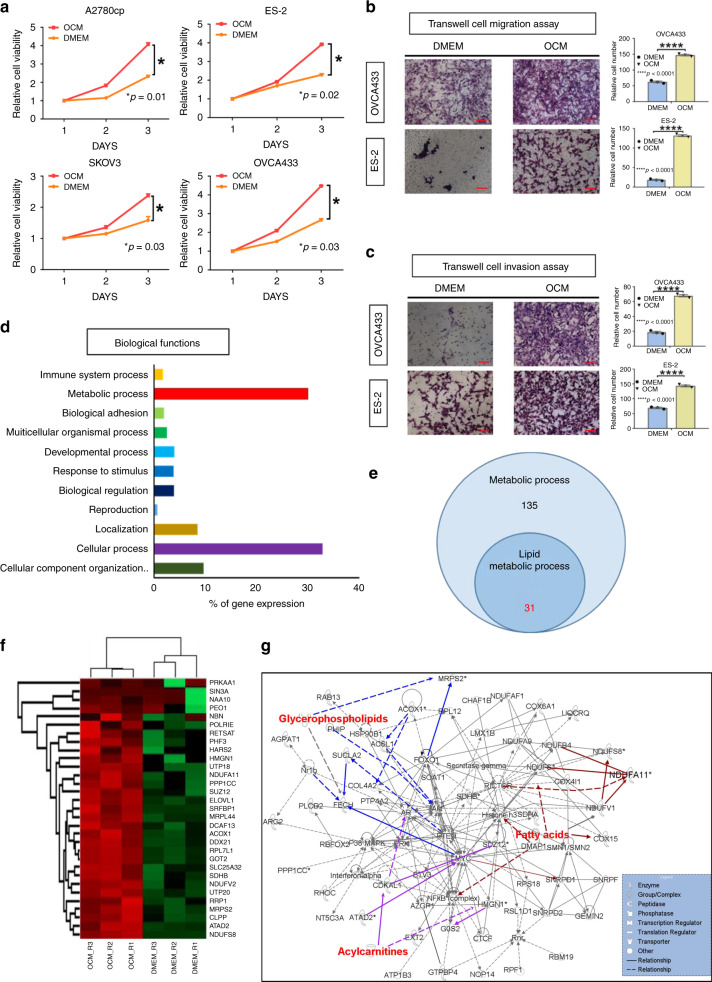
The lipid metabolic genes are frequently upregulated in ovarian cancer cells when cultured in OCM. **a** XTT cell proliferation assay demonstrates that treatment with OCM significantly increases cell growth in A2780cp, ES-2, SKOV3, and OVCA433 ovarian cancer cells. The relative cell viability was calculated by normalized to the mean value of day 1. **b** Transwell cell migration and **c** transwell cell invasion assays demonstrate that OCM treatment (12–24 h) promotes both cell migratory and invasive capacities in both OVCA433 and ES-2 cells. The stained cells were counted from four selected fields randomly. Representative images and quantitative results of cell migration and invasion were shown. Scale bar = 50 µm. **d** Ontology analysis on the altered genes detected by LC-MS/MS proteomic analysis indicates that most genes altered by OCM are associated with cellular and metabolic processes. The % of gene expression represents the % number of altered genes involved in each category of biological functions vs the total altered genes in ovarian cancer cells cultured in OCM as compared with the DMEM control. **e** A Venn diagram shows 31 out of 135 proteins related to metabolic processes are related to lipid metabolism. **f** Heatmap representation of the expression levels of the 31 proteins related to metabolism. **g** Ingenuity pathway analysis (IPA) depict interaction network of genes related to lipid metabolism in ovarian cancer cells. Results were presented as mean ± S.E.M. Data were analyzed by Student’s *t*-tests, and *p < 0.05 was considered as statistical significance

As the omentum is one of the preferred sites of ovarian cancer cell dissemination, it is of interest to study whether the omental microenvironment provides favorable conditions for ovarian cancer development and metastasis. Consistent with our previous finding^11^, ovarian cancer cells showed significant increases in cell proliferation when cultured in OCM (Fig. 1a). Similarly, these cancer cells exhibited increased cell migration and invasion rates in OCM (Fig. 1b, c). Using a liquid chromatography tandem-mass spectrometry (LC-MS/MS)-based label-free quantitative proteomics approach and gene ontology analyses, both cellular and metabolic processes are shown to be the most dominant biological functions in OCM-cultured ovarian cancer cells^12^ (Fig. 1d). Further analysis using Ingenuity pathway analysis (IPA) on metabolic interaction network revealed that 31 out of 135 upregulated (Fold Change > 1) metabolic targets were associated with lipid metabolism (Fig. 1e–g). These findings suggest that OCM provides a favorable microenvironment for ovarian cancer development and aggressiveness by increasing cellular and lipid metabolic activities.

### OCM provides free fatty acids for ATP production

**Fig. 2:**
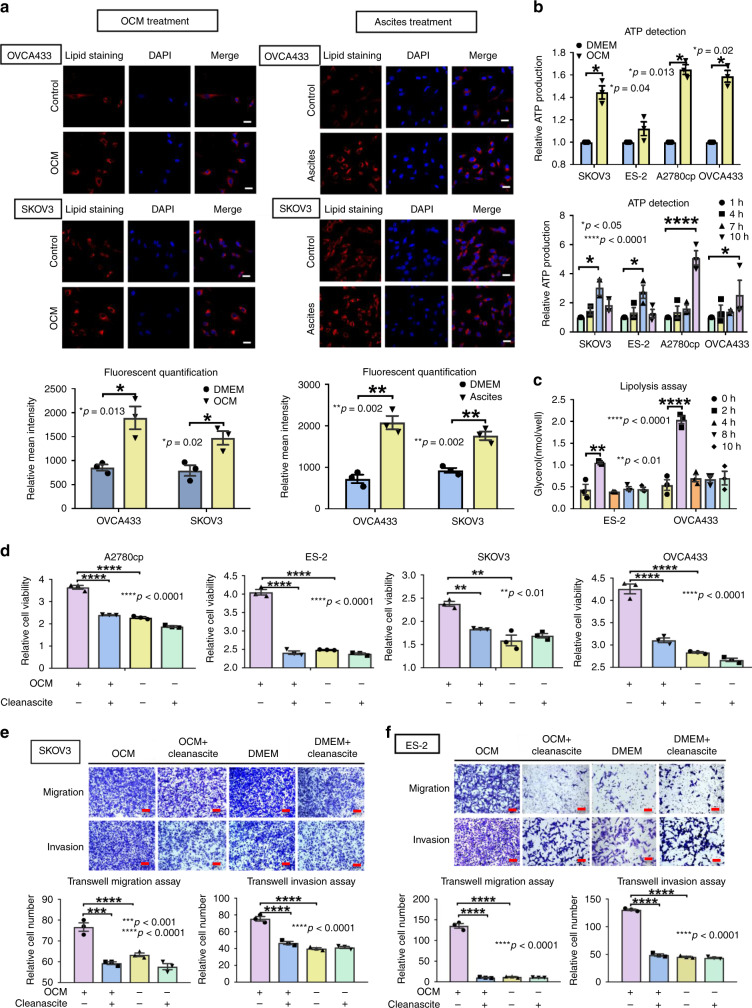
Free fatty acids from OCM provide an energy source for ovarian cancer cells. **a** Immunofluorescent and lipid staining analyses demonstrate that ovarian cancer cells SKOV3, OVCA433, A2780cp, and ES-2 exert lipogenesis by free fatty acid uptake from OCM (left panel) or ascites (right panel) and store free fatty acids as lipid droplets (red color by Nile Red staining) in their cytosol. Bar charts show the relative intensity of fluorescent signals (red) of OVCA433 (upper) and SKOV3 (lower) cells cultured in OCM or ascites (freshly obtained from ovarian cancer patients) for 24 h. DMEM (1%) was used as a negative control for both cell lines. Scale bar = 20 µm. **b** Spectrophotometric analysis and Luminescent ATP Detection Assay show a time-dependent increase in ATP production in SKOV3, ES-2, A2780cp, and OVCA433 cells. Each cell line exhibits the highest ATP production at different time points: SKOV3 and ES-2 for 7 h, while A2780cp and OVCA433 for 10 h. **c** Lipolysis Colorimetric Assay shows lipolysis activity from 0 to 10 h of ES-2 and OVCA433 cells upon culturing in OCM. **d** XTT cell proliferation assay shows that removal of fatty acids from OCM by Cleanascite (1:4 mixed with Cleanascite for 1 h before centrifugation) remarkably reduces the growth of ovarian cancer cells (A2780cp, ES-2, OVCA433, and SKOV3) compared with the negative control Cleanascite treatment in complete DMEM on day 3. Transwell cell migration/invasion assays demonstrate that the removal of free fatty acids by Cleanascite in OCM significantly reduces cell migration and invasion rates in (**e**) SKOV3 and (**f**) ES-2 cells, while the removal of fatty acids by Cleanascite in complete DMEM as negative controls does not change the cell migration or invasion rates of SKOV3 and ES-2 cells. The stained cells for cell migration and invasion were randomly counted from at least four selected fields. The representative images and bar charts were shown. Scale bar = 50 µm. Results were presented as mean ± S.E.M. Data were analyzed by Student’s *t*-tests or one-way/two-way ANOVA with Tukey’s post hoc test (*p < 0.05, **p < 0.01, ***p < 0.001, ****p < 0.0001)

To determine whether tumor cells utilize free fatty acids from OCM as the energy source to support tumor development and metastasis, we first examined the accumulation of lipid droplets in the cytoplasmic compartment using immunofluorescent and lipid staining analyses. The results suggested that ovarian cancer cells might take up free fatty acids from OCM or ascites, synthesize lipid droplets and store the fatty acids in their cytosol as early as 30 min to 1 h (Fig. 2a) after addition of the medium. Importantly, OCM functioned similarly to ascites such that OCM caused ovarian cancer cells to exhibit an ~0.85- to 2.8-fold increase in lipid droplets (Fig. 2a). Of note, luminescence assays for ATP showed that cellular levels of ATP in ovarian cancer cells were higher by up to 35% when cultured in OCM (Fig. 2b). SKOV3 and ES-2 cells showed an increase in ATP levels up to 7 h and then a decline up to 10 h, whereas OVCA433 and A2780cp cells showed continuous increases in ATP up to 10 h (Fig. 2b). These differences may be due to the different rates of ATP production in different cell contexts, different cell proliferation rates and/or different types of feedback loop controlling ATP production^13^. We found that ES-2 and OVCA433 cells commenced lipolysis and reached the maximal rate at 2 h of culture in OCM, which was followed by a reduction in lipolysis (Fig. 2c). Consistently, AMPK acts as a key energy sensor that maintains cellular energy homeostasis by controlling a number of physiological processes^14^. Western blot analysis revealed that the phosphorylation of AMPK at Thr172 became obviously elevated within the first 2–6 h, which was similar to the pattern of lipolysis (Fig. 2c) (Supplementary Fig. 1a) in ovarian cancer cells cultured in OCM. But ovarian cancer cells were cultured for a longer time (> 3 h), the intensity of phospho-AMPK^Thr172^ gradually decreased to levels close or even lower than the basal levels in ovarian cancer cells (Supplementary Fig. 1a), suggesting the reduction in AMPK phosphorylation was attributed to the negative feedback of high ATP production^15^. To determine whether fatty acids in OCM are the primary energy source, fatty acids from OCM was first removed by Cleanascite™ Lipid Removal Reagent (Supplementary Fig. 1b)^16^. Then, XTT cell proliferation assays showed that the growth rate of ovarian cancer cells was remarkably reduced in cells cultured in Cleanascite-treated OCM (Fig. 2d). Likewise, co-treatment with Cleanascite and OCM significantly attenuated the increased cell migration and invasion capacities of ES-2 and SKOV3 cells (Fig. 2e, f). These findings suggest that the fatty acid-enriched OCM provides as an energy source for supporting tumor growth and aggressiveness of ovarian cancer cells.

### Ovarian cancer cells exert metabolic reprogramming in OCM

**Fig. 3:**
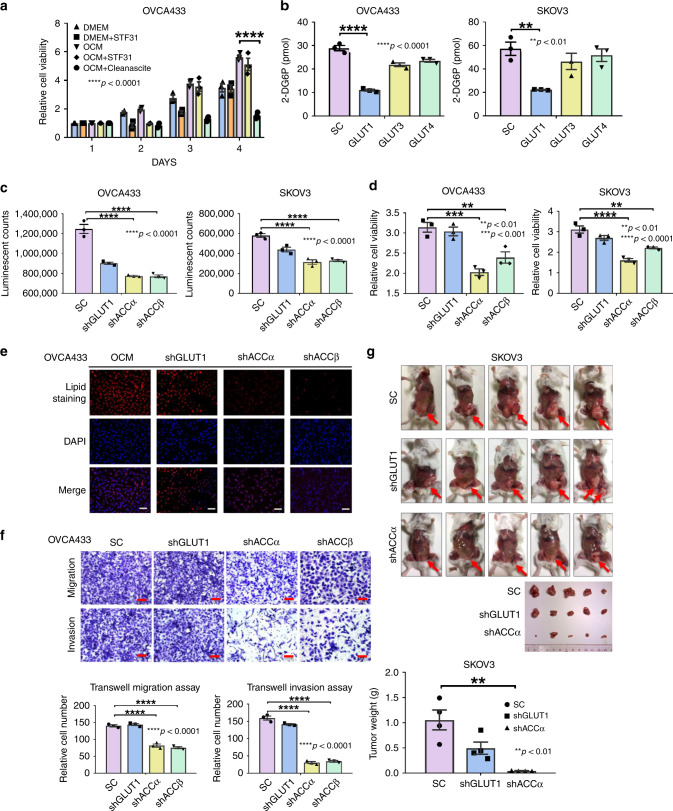
Ovarian cancer cells cultured undergo metabolic reprogramming in OCM. **a** XTT cell proliferation assay demonstrates that 3 days of co-treatment with a glucose uptake inhibitor, STF31 (5 μM), does not affect the growth of ovarian cancer cells cultured in OCM, whereas co-treatment of Cleanascite significantly attenuates the cell proliferation rate as compared with the effect of control OCM. **b** The uptake of glucose in OVCA433 and SKOV3 with stable knockdown of GLUT1, GLUT3, and GLUT4 by glucose uptake assay using 2-DG6P. **c** Spectrophotometric analysis and Luminescent ATP Detection Assay shows that stable knockdown of either ACCα or ACCβ significantly reduces ATP production in SKOV3 and OVCA433 cells, while knockdown of GLUT1 shows slight reduction (~15–21%) of ATP production in both cell lines. **d** XTT cell proliferation assay reveals that the cell proliferation of OVCA433 and SKOV3 cells with stably knockdown of GLUT1, ACCα, and ACCβ on day 3. **e** Immunofluorescent and lipid staining analyses show that the lipid droplet formation in OCM compared with DMEM control in OVCA433 cells with stably knockdown of GLUT1, ACCα, and ACCβ. Scale bar = 50 µm. **f** Transwell cell migration/invasion assays show that cell migration and invasion rates in OVCA433 cells with stably knockdown of GLUT1, ACCα, and ACCβ. The stained cells were counted randomly from at least four selected fields and the representative images with bar charts were shown. Scale bar = 50 µm. **g** Effects of GLUT1 or ACCα knockdown on ovarian cancer dissemination in xenograft mouse tumor model. SKOV3 cells with either GLUT1 (shGLUT1) or ACCα (shACCα) knockdown were injected into the intraperitoneal cavity of 5–6-week-old SCID female mice (*n* = 5). Scrambled control (SC) shRNA is used as a negative control (*n* = 5). Tumor nodule formation and localization are shown (red arrow); imaged were captured on day 45 after cancer cell inoculation. The bar chart indicates that the average tumor weight of the shACCα and shGLUT1 experimental groups are significantly lower than the SC groups. Results were presented as mean ± S.E.M. Data were analyzed by Student’s *t*-tests or one-way/two-way ANOVA with Tukey’s post hoc test (*p < 0.05, **p < 0.01, ***p < 0.001, ****p < 0.0001)

It is generally believed that the most prominent metabolic adaptation employed by cancer cells is enhanced glycolysis, known as the Warburg effect^17^. To examine whether ovarian cancer cells undergo metabolic reprogramming when cultured in OCM or fatty acid-enriched medium, we first used STF31, an inhibitor of the glucose transporter GLUT1, to inhibit the use of glycolysis to generate energy for supporting ovarian cancer cell growth. XTT cell proliferation assays showed that co-treatment with STF31 led to an initial decline in cell proliferation of ovarian cancer cells, but that long-term culture could restore the cell proliferation rate despite a slightly lower cell proliferation rate than that of cells cultured in OCM (Fig. 3a). In addition, when OCM was co-treated with Cleanascite, the cell proliferation rate was completely impaired as compared with the control OCM (Fig. 3a). To avoid the concerns of off-target effects of STF31 and Cleanascite, the key glycolytic gene, *SLC2A1* (encoding GLUT1) was stably knocked down using lentiviral shRNAi in OVCA433 and SKOV3 cells. Depletion of GLUT1 did not alter expression of other GLUT isoforms, such as GLUT3 and GLUT4 (Supplementary Fig. 2a). Besides, knockdown of GLUT1 reduced glucose uptake (measured using 2-deoxyglucose) by 65% compared with scrambled control (SC), while the same cells with stably knockdown of GLUT3 and GLUT4 had no change in glucose uptake capacities (Fig. 3b) (Supplementary Fig. 2a), suggesting that GLUT1 is the primary glucose transporter in ovarian cancer cells.

To confirm the importance of lipolysis and lipogenesis in ATP production, we investigated the role of acetyl-CoA carboxylase (ACC) because it consists of two isoforms: ACCα, which locates in the cytoplasm, is involved in lipogenesis, and ACCβ, which locates at the mitochondrial membrane, controls fatty acid oxidation^18^. Using lentiviral shRNAi, both ACCα and ACCβ were stably knocked down in SKOV3 and OVCA433 cells (Supplementary Fig. 2b). Interestingly, while depletion of GLUT1 has only a small effect (15–21%) on ATP levels in SKOV3 or OVCA433 cells cultured in OCM, depletion of either ACCα or ACCβ resulted in much larger effects (40 to 50%) in both lines (Fig. 3c). This indicates that ACCα and ACCβ play more important roles than GLUT1 in lipogenesis (ACCα) and ATP production (ACCβ) of ovarian cancer cells cultured in OCM. Functionally, while the XTT cell proliferation assay showed that knockdown of GLUT1 initially influenced cell growth, there was almost no inhibitory effect on cell growth after day 3 or day 4 (Fig. 3d). By contrast, knockdown of ACCα markedly impaired proliferation of both cell lines by 1.6–2-fold (Fig. 3d). Similarly, knockdown of GLUT1 had no effect on lipid droplets, whereas depletion of ACCα obviously reduced the lipid droplet formation in OVCA433 and SKOV3 (Fig. 3e) (Supplementary Fig. 2c). Moreover, knockdown of ACCα obviously inhibited the capacities of cell migration and invasion in ovarian cancer cells, whereas knockdown of GLUT1 had no effect on cell migration/invasion in ovarian cancer cells (Fig. 3f) (Supplementary Fig. 2d). Using an in vivo tumor xenograft mouse model, we also demonstrated that knockdown of ACCα reduced 95%, and more than three-fold of tumor weight when compared with SC and  knockdown of GLUT1 in SKOV3 cells, respectively (Fig. 3g). Taken together, these results indicate that ovarian cancer cells cultured in OCM or ascites undergo metabolic reprogramming, switching from the use of aerobic glycolysis to the use of lipid metabolism to produce ATP for their tumorigenic capacities.

### Ovarian cancer cells undergo lipogenesis in OCM

**Fig. 4:**
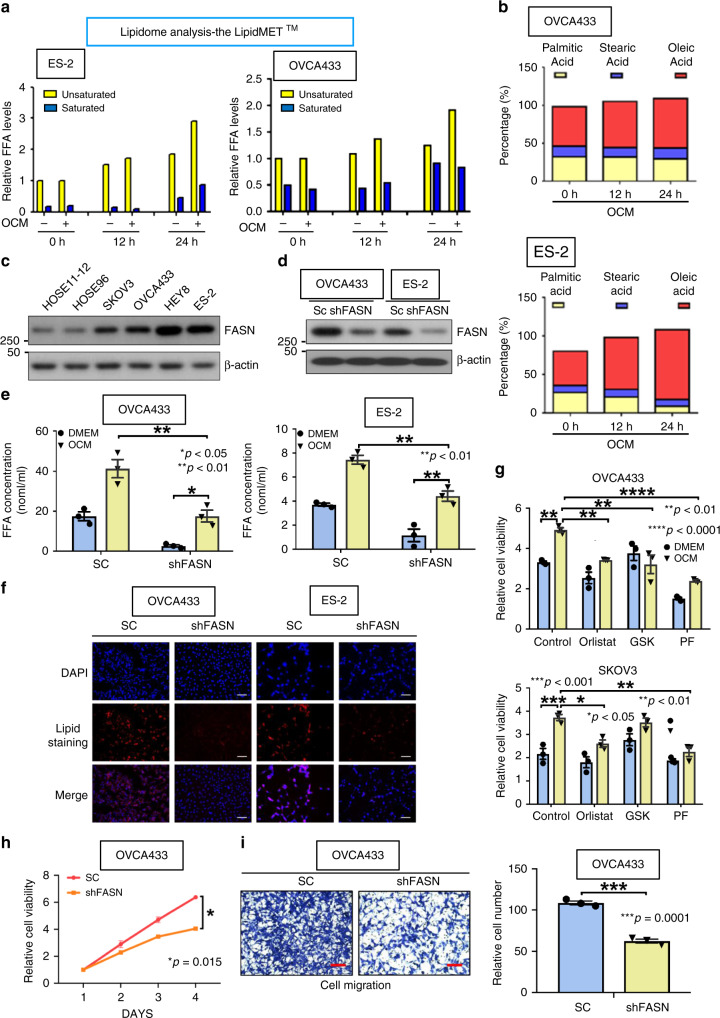
Ovarian cancer cells undergo lipogenesis in OCM. **a** The bar chart shows the Lipidomic analysis of the amount of intercellular unsaturated and saturated fatty acids in two ovarian cancer cells which were cultured in OCM from 0 to 24 h. The DMEM is used as a negative control. The Lipidomic analysis was performed by Metabo-Profile, Shanghai, China. **b** The percentage bar chart shows the changes of the major unsaturated fatty acid, Oleic acid, and two saturated fatty acids, Stearic acid and Palmitic acids in OVCA433 and ES-2 cells cultured in OCM from 0 to 24 h. **c** Western blot analysis compares the expression level of a key lipogenic enzyme, FASN, in two HOSEs and four ovarian cancer cell lines, SKOV3, OVCA433, Hey8, and ES-2. **d** Western blot analysis shows the reduction of FASN knockdown by lentiviral shRNAi approach in OVCA433 and ES-2. **e** Free fatty acid assay demonstrates the depletion of FASN leads to the reduction of long chain fatty acid (>8 carbon) in OVCA433 and ES-2 cells when cultured in DMEM or OCM. **f** Lipid droplet formation assay reveals the formation of lipid droplets in FASN knockdown OVCA433 and ES-2 cells as compared with their scrambled controls (SC). Scale bar = 50 µm. **g** XTT cell proliferation assay shows that 3 days of co-treatment with the above FASN inhibitors, orlistat (30 µM) and GSK2194069 (100 nM) (GSK), or AMPK activator, PF-06409577 (50 μM) (PF), significantly reduces the OCM-induced cell growth rate in SKOV3 and OVCA433 cells. **h** XTT cell proliferation assay reveals that the cell proliferation of OVCA433 with or without FASN knockdown co-cultured in OCM for 4 days. **i** Transwell cell migration assay shows the cell migratory rate of OVCA433 with or without FASN knockdown co-cultured in OCM for 12 h. The stained cells were counted from three selected fields randomly. Representative images and quantitative results of cell migration were shown. Scale bar = 50 µm. Results were presented as mean ± S.E.M. Data were analyzed by Student’s *t*-tests or one-way/two-way ANOVA with Tukey’s post hoc test (*p < 0.05, **p < 0.01, ***p < 0.001, ****p < 0.0001)

To better understand how ovarian cancer cells utilized the absorptive fatty acids from OCM, we analyzed the composition of fatty acids in OCM/ascites and ovarian cancer cells by Lipidomic profiling. Results indicated there was an higher accumulation of unsaturated fatty acids (UFA) than  saturated fatty acids (SFA) in ovarian cancer cells when cultured in OCM for 24 h (Fig. 4a) Consistently, both OCM and ascites exhibited a higher ratio of UFA to SFA (Supplementary Fig. 3a). Of note, the amount of major UFA oleic acid increased much faster than the SFAs such as stearic acid and palmitic acid decreased from 0 to 24 h in OCM (Fig. 4b), while the amounts of oleic acid, palmitic acid, and linoleic acid were equally distributed in OCM or ascites (Supplementary Fig. 3b). This indicates OCM-cultured ovarian cancer cells has increased activity in biosynthesis of long chain fatty acids for formation of lipid droplets. Fatty acid synthase (FASN) is a key enzyme of biosynthesis of fatty acids^19,20^, and western blotting clearly showed that the levels of FASN in rapidly growing ovarian cancer cell lines were much higher than the human immortalized normal ovarian epithelial cells (HOSEs) (Fig. 4c), that is consistent with other cancer reports^19,20^. Depletion of FASN in ovarian cancer cells by shRNAi caused the reduced content of long chain fatty acids in both OCM and DMEM by twofolds when compared with the scrambled controls (SC) (Fig. 4d, e). Consistently, the depletion of FASN also led to remarkable reduction of lipid droplet formation in ovarian cancer cells (Fig. 4f), supporting the important role of FASN in lipogenesis of ovarian cancer cells^21^. To further confirm that activated FASN and the suppressed phospho-AMPK^Thr172^ due to high ATP production are responsible for enhancing ovarian cancer oncogenic properties in OCM, we added two potent FASN-specific inhibitors (orlistat and GSK2194069 (GSK)) or an AMPK potent activator (PF-06409577 (PF)) to ovarian cancer cells in OCM. These inhibitors completely impaired the increased cell proliferation rate mediated by OCM in ovarian cancer cells (Fig. 4g). Consistently, the depletion of FASN caused a remarkable reduction of cell proliferation, cell migration, and invasion of ovarian cancer cells in OCM (Fig. 4h, i and Supplementary Fig. 4), indicating FASN is another key target for the oncogenic properties of ovarian cancer cells in OCM. Taken together, these findings have suggested that ovarian cancer cells utilize absorbed fatty acids for ATP production and synthesize de novo fatty acids used as cellular components to support their increased cell growth in OCM.

### Downstream AMPK-regulated oncogenic signaling

**Fig. 5:**
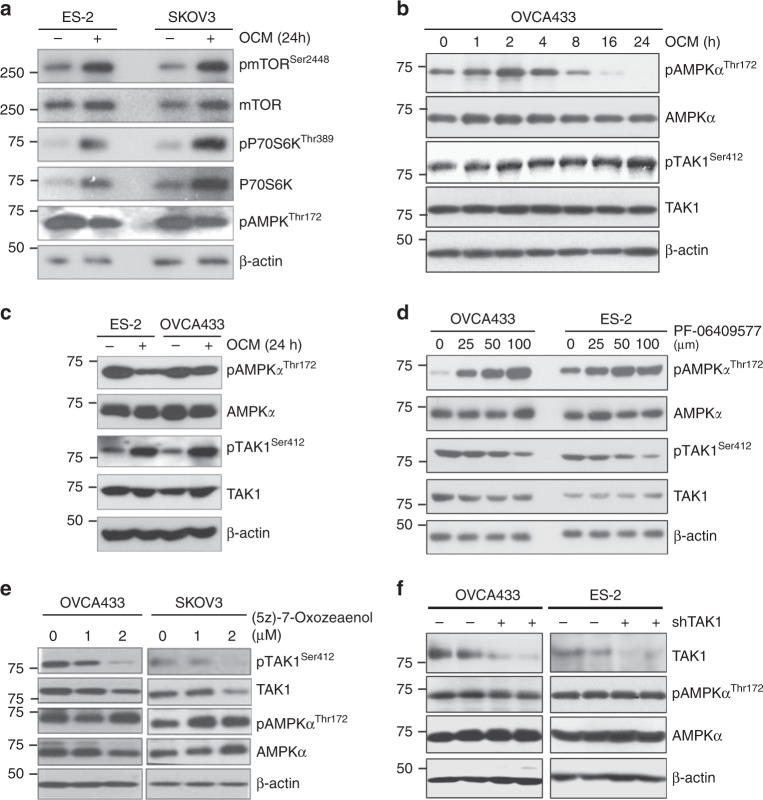
Oncogenic pathways regulated by AMPK in ovarian cancer cells. **a** Long-term culture with OCM (24 h) leads to reduction in AMPK activity (pAMPK^Thr172^), activation of mTOR activity (pmTOR^Ser2448^), and increased phosphorylation of p70S6K (pP70S6K^Thr389^) in ES-2 and SKOV3 cells. **b** While culturing OVCA433 cells in OCM for 24 h leads to downregulation of pAMPK^Thr172^, but elevation of the level of pTAK^Ser412^. **c** Long-term culture with OCM (24 h) with ES-2 and OVCA433 causes an inverse relationship between the AMPK (reduced pAMPK ^Thr172^) and TAK1 (increased pTAK1^Ser412^) activities. **d** The level of pAMPK^Thr172^ is increased, while the level of pTAK1^Ser412^ is reduced upon treatment with AMPK activator, PF-06409577 (24 h), in a dose-dependent manner in OVCA433 and ES-2 ovarian cancer cells. **e** Co-treatment with TAK1 inhibitor, (5Z)-7-Oxozeaenol (2.5 μM), substantially inhibits the expression of pTAK1^Ser412^ in a dose-dependent manner (24 h), and this effect is accompanied by an increase in pAMPK^Thr172^ levels in OVCA433 and SKOV3 cells. **f** Knockdown of endogenous TAK1 by shRNAi more than 70% does not alter either pAMPK^Thr172^ or total AMPK in OVCA433 cells

Activated AMPK has been shown to impede cell growth and proliferation in part by inhibiting mTORC1 signaling^22^. In this study, we have shown that AMPK activation was reduced in the presence of OCM due to negative feedback by high ATP content. To determine whether reduced AMPK activity leads to increased oncogenic capacity of ovarian cancer cells, we examined mTORC1 signaling. After 24 h of culture in OCM, the phosphorylation of AMPK at Thr172 was reduced and was inversely correlated with the increased phosphorylation of mTOR at Ser2448 and phospho-pP70S6K Thr389 in ES-2 and SKOV3 ovarian cancer cells after culturing in OCM for 24 h (Fig. 5a). This suggests that the reduced AMPK activity induced activation of mTOR signaling in ovarian cancer cells. However, mTOR signaling could partially explain the enhanced aggressiveness of ovarian cancer cells^23,24^. We previously reported that increased TAK1 activity, through phosphorylation of Ser412, is required for the activation of the NF-κB signaling cascade in mediating ovarian cancer metastasis upon treatment with OCM^11^. Coincidentally, we found that the reduced level of phospho-AMPK^Thr172^ inversely correlated with the increased level of phospho-TAK1^Ser412^ in OVCA433 and ES-2 ovarian cancer cells when cultured in OCM for 24 h (Fig. 5b, c). This result reveals that the reduced AMPK activity favors the activities of TAK1 and NF-κB in promoting ovarian cancer cell aggressiveness. On the other hand, upon treatment with 6-Chloro-5-[4-(1-hydroxycyclobutyl)phenyl]-1H-indole-3-carboxylic Acid (PF-06409577), a potent and direct AMPK activator^25^, we demonstrated that increased AMPK inversely correlated with TAK1 activity of ovarian cancer cells in a dose-dependent manner of PF-06409577 (Fig. 5d). To examine whether TAK1 alters AMPK activity, ovarian cancer cells were co-treated with TAK1 inhibitor 5Z-7-Oxozeaenol (5Z-O), and results showed that inhibition of TAK1 activity in a dose-dependent manner of 5Z-O slightly enhanced AMPK phosphorylation in SKOV3 only, while there’s no effect on AMPK phosphorylation in OVCA433 and SKOV3 cells (Fig. 5e). The slight increase in AMPK activity might be due to the cytotoxicity of 5Z-O in SKOV3 but not in OVCA433 cells. To address this question, TAK1 in OVCA433 was knockdown by shRNA approach, while there was no change in either phospho-AMPK^Thr172^ or total AMPK levels (Fig. 5f), suggesting AMPK is upstream rather than downstream of TAK1 in ovarian cancer cells. Taken together, the reduced AMPK activity resulting from high ATP content led to activation of both mTORC1 signaling and TAK1/NF-κB signaling in ovarian cancer cells cultured in OCM.

### Targeting AMPK and TAK1 impair ovarian cancer aggressiveness

**Fig. 6:**
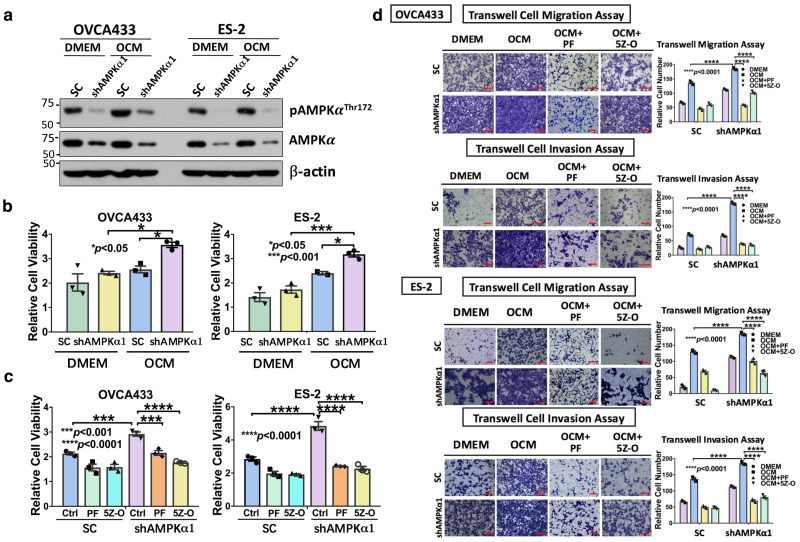
Targeting AMPK or inhibiting TAK1 signaling activity impairs ovarian cancer aggressiveness. **a** AMPKα1 knockdown stable clones were established via shRNA lentiviral approach targeting the α1-isoform of AMPK in OVCA433 and ES-2 cells, and cells cultured in DMEM or OCM (2 h) could not induce AMPK activity (pAMPK^Thr172^) in shAMPKα1 clones compared with their scrambled controls (SC) of both cell lines. **b** XTT cell proliferation assay shows that OCM can promote proliferation of ES-2 and OVCA433 cells, but after depletion of AMPKα1 in both cell lines, shAMPKα1 clones show significantly higher cell proliferation than that of scrambled controls (SC) cultured in DMEM or OCM for 3 days. **c** XTT cell proliferation assay shows that 3 days of co-treatment with AMPK activator PF-06409577 (50 μM) (PF) or TAK1 inhibitor (5Z)-7-Oxozeaenol (2.5 μM) (5Z-O) significantly inhibited cell proliferation in both parental and AMPKα knockdown clones compared with the scrambled controls (SC) of ES-2 and OVCA433 cells. **d** Transwell cell migration/invasion assays demonstrate that the cell migratory and invasive capacities of OVCA433 and ES-2 cells are enhanced by OCM. Knockdown of AMPKα1 further promotes the migration and invasion rates of cells cultured in OCM or DMEM. In contrast, co-treatment with AMPK activator PF-06409577 (50 μM) (PF) or TAK1 inhibitor 5Z-O (2.5 μM) somewhat abrogated the cell migratory and invasive capacities of AMPKα1 knockdown clones and scrambled controls (SC) of both cell lines. The stained cells were counted at least from four randomly selected fields. Representative images and quantitative results were shown by bar charts. Scale bar = 50 μm

Given that AMPK acts as an upstream effector of TAK1/NF-κB signaling, we next investigated whether targeting AMPK alone, or in combination with TAK1 suppression could inhibit the oncogenic capacities of ovarian cancer cells cultured in OCM. Knockdown of AMPKα1 impaired the activation of AMPK activity in OCM-cultured ovarian cancer cells with different levels due to varied cell context (Fig. 6a). In addition, knockdown of AMPKα1 favored the growth of ovarian cancer cells in OCM compared with DMEM control (Fig. 6b). However, co-treatment with PF-06409577 could abrogate the increased cell proliferation caused by AMPKα1 knockdown in ovarian cancer cells in OCM (Fig. 6c). Consistently, co-treatment with TAK1 inhibitor, 5Z-O, in AMPK-knockdown ovarian cancer cells resulted in suppression of cell proliferation, which was similar to the effect of AMPK activator PF-06409577 (Fig. 6c). To better assess the cellular responses to drug treatments, we performed 3D spheroid ovarian cancer cell culture for OVCA433 and ES-2 and co-treated with PF-06409577, Orlistat and 5Z-O alone at serial diluted concentrations or in combination. Using CellTiter-Glo® 3D cell viability assay, the IC50 values of single drug were calculated by nonlinear regression, while the combination index (CI) for evaluating the drug synergy effect of the drug combination was performed by using the Chou and Talalay method in CalcuSyn software (Biosoft, Version 2.1) (Supplementary Fig. 5a). The CI values of combined PF-06409577 and Orlistat or 5Z-O in both cell lines ranged from 0.124 to 0.641, suggesting the above combined drugs exhibited synergistic anti-cancer effect on ovarian cancer cells (Supplementary Fig. 5b). Moreover, using transwell cell migration and invasion assays, knockdown of AMPKα1 in ES-2 and OVCA433 cells further enhanced their migratory and invasive capacities by two- to sixfold in OCM (Fig. 6d). In contrast, co-treatment with either PF-06409577 or the TAK1 inhibitor 5Z-O attenuated the increased cell migratory and invasive capacities caused by AMPKα1 knockdown in ovarian cancer cells (Fig. 6d). These results suggest that pharmaceutical activation of AMPK is equivalent to suppressing the TAK1/NF-κB signaling activity in driving ovarian cancer cell aggressiveness in OCM.

### AMPK/TAK1/NF-κB signaling is required for metastatic seeding

**Fig. 7:**
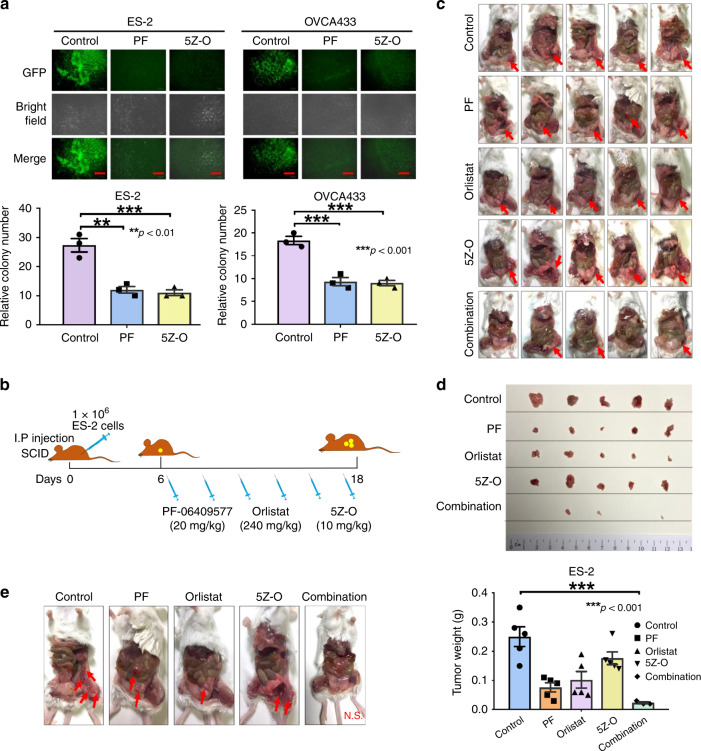
AMPK/FASN/TAK1/NF-κB signaling axis is required for metastatic colonization. **a** eGFP-labeled ES-2 and OVCA433 ovarian cancer cells were established by infection with LV-CMV-RLuc-IRES-GFP lentiviral particles. After incubating GFP-labeled ovarian cancer cells with omental tissues from 6- to 8-week-old SCID female mice, the results show that ES-2 and OVCA433 cells exhibit a significant number of tumor colonies on murine omenta on day 30. However, co-treatment with AMPK activator PF-06409577 (50 μM) (PF) or TAK1 inhibitor 5Z-O (2.5 μM), remarkably reduces the number and size of tumor colonies by 45–55% on the murine omenta. Scale bar = 100 μm. **b** Schematic overview showing the experimental protocol of the anti-tumorigenic effect of the combined cocktail of the AMPK activator PF-06409577 (20 mg/kg), FASN inhibitor orlistat (240 mg/kg), and TAK1 inhibitor 5Z-O (10 mg/kg) on ovarian cancer cells in SCID mice. ES-2 cells (1 × 10^6^/200 µl) were injected into the intraperitoneal cavity of 5–6-week-old SCID female mice. On day 6, the above three drug reagents were injected individually or in combination (for a total of six injections) from day 6 to day 18. **c** Images show tumor formation in all mice. **d** Images showing tumor nodules obtained from all mice. The bar chart shows the average tumor weight obtained from each group. **e** Representative images showing the number and locations of tumor nodules distributed in the intraperitoneal cavity of each mouse group. Results were presented as mean ± S.E.M. Data were analyzed by one-way ANOVA with Tukey’s post hoc test (*p < 0.05, **p < 0.01, ***p < 0.001, ****p < 0.0001)

To further investigate whether this AMPK/TAK1/NF-κB signaling axis is required for promoting metastatic seeding of ovarian cancer cells in omental tissues, we established an ex vivo murine omental system by culturing omenta from 6- to 8-week-old SCID female mice (to exclude immune cells)^5^. Two highly metastatic ovarian cancer cells (ES-2 and OVCA433) were eGFP-labeled and were added to the omental culture system. The results showed that co-treatment of either the AMPK activator PF-06409577 (PF) or the TAK1 inhibitor 5Z-O could significantly inhibit tumor colony formation on the murine omenta (Fig. 7a), suggesting that the activation of AMPK or inhibition of TAK1/NF-κB signaling could be efficient for inhibiting tumor colonization in the ex vivo omental model. We next investigated whether targeting AMPK/ACC/FASN-mediated lipogenesis and the AMPK/TAK1/NF-κB signaling cascade suppresses ovarian cancer cell metastasis in vivo using an intraperitoneal xenograft SCID mouse model. Six days after intraperitoneal injection of ES-2 into 5–6 weeks SCID female mice, AMPK activator (PF-06409577, 20 mg/kg), FASN inhibitor (orlistat, 240 mg/kg), and TAK1 inhibitor (5Z-O, 10 mg/kg) were i.p. injected alone or as a combined cocktail for every 3 days (total of six injections) (Fig. 7b). As ES-2 is an aggressive ovarian cancer cell, it form tumors in a very short time (within 3 weeks). In the control, PF-06409577, orlistat and 5Z-O groups, five out of five mice formed tumors, while only three out of five mice had small tumor nodules in the combined cocktail group (Fig. 7c). Of note, all mice in the negative group had ascites formation, while the other groups had only 1–2 mice with ascites. Analysis of tumor weight showed that tumor-bearing mice had 70%, 60%, and 30% tumor reduction following treatment with PF-06409577, orlistat, and 5Z-O, respectively (Fig. 7d). Importantly, the combined cocktail could further reduce tumor formation up to 95% (Fig. 7d). In addition, co-treatment with either PF-06409577, orlistat or 5Z-O could suppress the number of tumor nodules compared with the negative control (Fig. 7e). As expected, the combined cocktail could completely abrogate tumor dissemination of ovarian cancer cells in the intraperitoneal cavity of mice (Fig. 7e). More importantly, by blood test, treatment with the above drugs alone or together did not cause any damage to the liver or renal functions (Table 1). Taken together, both the ex vivo omental system and in vivo tumor xenograft mouse model support our notion that targeting AMPK/ACC/FASN for lipogenesis and AMPK/TAK1/NF-κB oncogenic signaling is able to inhibit metastatic dissemination of ovarian cancer cells in the peritoneal cavity.

**Table 1:** Table showing the renal and liver function tests of two groups of SCID mice and control and drug combined cocktail tests

Animal	Control	Drug combination	Reference range
*Liver function*			
Bilirubin total	0.7	0.6	0.1–0.9 mg/dL
Bilirubin direct	0.6	0.6	0.1–1.2 mg/dL
Alkaline phosphatase	200	122	62–209 U/L
Protein total	5.2	4.9	3.6–3.3 mg/dL
ALT	45	54	28–132 U/L
AST	76	100	59–247 U/L
*Renal function*			
Albumin	2.8	2.5	2.5–4.8 g/dL
Calcium	10	10.2	5.9–9.4 mg/dL
Chloride	113	110	92–120 mmol/L
Creatinine	<0.30	<0.30	0.20–0.80 mg/dL
Glucose	112	141	90–192 mg/dL
Phosphorus	10.3	10.3	6.1–10.1 mg/dL
Potassium	6.5	6.5	4.6–8.0 mmol/L
Sodium	148	144	124–174 mmol/L
BUN	19.5	16	18–29 mg/dL

## Discussion

Peritoneal metastases are common, and are associated with poor prognosis, in advanced gynecological and gastrointestinal cancers^26^. Of the different routes of peritoneal metastases, omental metastasis is the most common in early presentation of ovarian cancers^27^. Recent evidence has suggested that adipocytes, the major cell population of the omentum, fuel ovarian cancer cell growth and aggressiveness in colonization^9^, thus supporting the rationale for why cancer cells prefer to metastasize in lipid-enriched microenvironments or niches^28^. In this study, we established OCM to mimic the tumor microenvironment of the intraperitoneal cavity to study its effect on tumor metabolism and oncogenic capacities^11^. Our results revealed that ovarian cancer cells undergo metabolic reprogramming by shifting from aerobic glycolysis to fatty acid oxidation to produce ATP and thus support their increased cell proliferation and enhanced cell migration/invasion capacities in the fatty acid-enriched OCM. We further found that AMPK acts as a key energy regulator in controlling ACC in lipogenesis and elevated lipolysis and β-oxidation activities. On the other hand, the high ATP production from β-oxidation suppressed AMPK activity, which in turn, liberated the inhibition of mTOR activity and promoted cancer cell growth^29^. Importantly, we are the first to demonstrate that TAK1/NF-κB signaling, as the dominant downstream signaling pathway, is similar to mTOR signaling, inversely modulated by AMPK activity, and involved in ovarian cancer aggressiveness in the intraperitoneal cavity or ascites microenvironment.

Metabolic reprogramming has been recently recognized as another hallmark of cancer because it is critical for cancer metastatic progression and minimal residual disease^30^. Tumor tissues are known to be composed of not only cancer cells but also other cell types, indicating that cancer cells are surrounded by heterogeneous intratumoral backgrounds^31^. Thus, genetic/epigenetic alterations during tumor development cause cancer cells to possess the capacity of cellular metabolic heterogeneity or metabolic symbiosis in order to adapt and develop during drastic changes in the nutrient microenvironment^32^. The Warburg effect is a distinct feature of many human cancers that allows cells to exhibit enhanced aerobic glycolysis to generate energy for their rapid cell proliferation under stress conditions^33^. However, ascites provide a distinct tumor microenvironment that affects ovarian cancer cells in different aspects and associates with peritoneal metastases and poor prognosis^34^. Malignant ascites act as a reservoir of a complex mixture of bioactive lipids, pro-inflammatory factors and a tumor-promoting microenvironment for metastatic progression of ovarian cancer cells^35,36^. The high free fatty acid content in ascitic fluid provides a huge energy source and may force ovarian cancer cells to undergo metabolic reprogramming from aerobic glycolysis to fatty acid β-oxidation to produce energy for tumor growth. Indeed, our findings showed that ovarian cancer cells conduct lipogenesis and fatty acid β-oxidation for cellular blocks synthesis and ATP production. On the other hand, the inhibition of glycolysis using STF31^37^, or shRNAi targeted at GLUT1^38^, could not alter ovarian cancer cell proliferation or cell migration/invasion when co-cultured in OCM. By contrast, knockdown of ACCα and/or ACCβ not only impaired lipogenesis but also fatty acid β-oxidation for ovarian cancer cell proliferation and cell migration/invasion of ovarian cancer cells cultured in OCM. These data indicate the metabolic plasticity of ovarian cancer cells in the adaptation of abundant availability of free fatty acids in ascites or omental tissues induce ovarian cancer cells to shift glycolysis to lipid metabolism for ATP production to sustain their cell survival and tumor progression^39,40^.

AMPK is a key energy sensor that not only maintains the balance of energy consumption and expenditure but also modulates cell protection against stresses and cell proliferation^41,42^. Thus, AMPK activity should be increased in ovarian cancer cells under the hypoxic ascites microenvironment because of pathogenic stresses and low oxygen content. However, it is well known that sustained high AMPK activity may inhibit mTOR signaling in tumor growth^43^. In fact, as cancer cells prefer low AMPK for tumor development and metastasis, low AMPK levels commonly occur in advanced cancer cells because genetic/epigenetic progression enhances cell tolerance to stress and increases cell growth and aggressiveness^44,45^. Our study showed that ovarian cancer cells initially displayed high AMPK activity because of the change in the culture conditions from DMEM to a fatty acid-enriched medium. The high AMPK activity could protect cells against stresses from different media; however, importantly, switching to lipogenesis and lipolysis could have also had a protective effect on ovarian cancer cells. Interestingly, we found that there is an inverse relationship between ATP production and AMPK activity in ovarian cancer cells, suggesting that there is a negative feedback loop to suppress AMPK activity after prolonged culture in OCM^43^. As a result, the combination of high ATP and low AMPK activity synergistically enhances ovarian cancer aggressiveness in OCM. Previous investigations have noted that low AMPK activity favors tumor progression and invasion^46,47^. Consistently, our findings using knockdown of AMPKα in ovarian cancer cells confirmed that low AMPK activity is required for ex vivo and in vivo tumor growth.

Emerging evidence has suggested AMPK modulates numerous downstream signaling pathways for cell survival and other properties^48,49^. This indicates the suppression of mTOR signaling is not sufficient to account for the effect of low AMPK in promoting ovarian cancer aggressiveness. Indeed, we previously reported that TAK1/NF-κB signaling plays a critical role in enhancing ovarian cancer tumorigenicity in OCM^11^. Although it’s controversial that TAK1 may act as the upstream or downstream target of AMPK^50,51^, our findings have confirmed that TAK1/NF-κB signaling, in line with mTORC1, is negatively regulated by AMPK in ovarian cancer cells. Therefore, inhibition of TAK1/NF-κB signaling by 5Z-O or suppression of AMPK/ACC/FASN signaling in lipogenesis by the potent AMPK activator PF-06409577^52^ or orlistat could inhibit ovarian cancer dissemination and tumor growth in vivo. Importantly, we found that a combined cocktail of the above reagents could further exert synergistic anti-cancer effects in 3D spheroids culture in vitro and metastatic dissemination in vivo. Previous studies have shown that low dosage of PF-06409577^25^, orlistat^53^, and 5Z-O^54^ exhibit low toxicity in vitro and in vivo. Indeed, a blood test for renal and liver functions in mice that received the same dose and time intervals of the above reagents revealed no sign of toxicity in the mice. This indicates these reagents exert no toxicity in vivo, at least in low doses.

In summary, dynamic metabolic alterations occur in advanced stage ovarian cancer cells to adapt to drastic changes in the nutrient microenvironment. In this study, ovarian cancer cells utilize lipid metabolism in the ascites or omental microenvironment during metastatic progression through AMPK/ACC/FASN-mediated lipogenesis and AMPK/TAK1/NF-κB signaling cascades. Hence, targeting these pathways by a combined cocktail of low-toxic compounds may be an alternative therapeutic intervention to impede ovarian cancer peritoneal metastases.

## Methods

### Ovarian cancer samples and cell lines

Ovarian cancer cell lines A2780cp and SKOV3 (high-grade ovarian cancer cell lines) and ES-2 (kindly provided by Prof. Benjamin Tsang, University of Ottawa), Hey8 (Prof. Alice Wong, The University of Hong Kong) and OVCA433 (Prof. George Tsao, The University of Hong Kong) (high-grade serous subtype)^55,56^ were all cultured in Dulbecco’s-modified Eagle medium (DMEM) (Gibco-BRL, Minneapolis, MN, USA) supplemented with 10% heat-inactivated fetal bovine serum (FBS) (Gibco-BRL) and penicillin/streptomycin (100 units/mL); cells were incubated at 37 °C with an atmosphere of 5% CO_2_. In-house short tandem repeat (STR) DNA profiling analysis was used to authenticate the above cell lines, and mycoplasma contamination was also assessed. Freshly collected peritoneal fluids or ascites, ovarian tumor specimens and omental tissues were surgically resected from ovarian cancer patients of Queen Mary Hospital. The use of these clinical samples in this study was approved by the Institutional Review Board of the University of Hong Kong/Hospital Authority Hong Kong West Cluster (HKU/HA HKW IRS) (IRS Reference Number: UW 11-298).

### OCM and commercial kits

Freshly resected omental tissues from ovarian cancer patients were directly collected during the operation at Queen Mary Hospital and delivered to our laboratory within 2 h. The omental tissue was washed in PBS and minced into pieces at similar size. After transferring into 1% DMEM, omental mixture was incubated at 37 °C with 5% CO_2_ for 24 h. Then all the omental tissue was removed on the following day by a SPL cell strainer (Bio Lab, Conyers, GA, USA), followed by centrifugation at 1500 × *g* for 5 min for three times. Finally, the OCM was accomplished after filtration the supernatant medium 0.7 μm column filter. For storage, e.g., 1 month, OCM was aliquoted and stored at 4 °C for further study. Cleanascite™ Lipid Removal Reagent (Biotech Support Group, Monmouth Junction, NJ, USA) was used for selectively removing lipoproteins, lipids, floating fats, and cell debris without affecting other serum components including hormones in OCM or ascites. 2-DG (equivalent to glucose) was taken up by glucose transporters and metabolized to 2-DG-6-phosphate (2-DG6P). The other kits and drugs used in this study are shown in Supplementary Table 1.

### Stable cell transfection and cell sorting

Stable knockdown clones for AMPKα, GLUT1, GLUT3, GLUT4, ACCα, and ACCβ of ovarian cancer cells were established by lentiviral shRNAi-mediated particles (Santa Cruz, Dallas, Texas, USA) and selected with 1 μg/10 mL puromycin for 2 weeks. The efficiency of transfection was verified by western blot analysis. To achieve eGFP-labeling cells, LV-CMV-RLuc-IRES-GFP pre-made lentiviral particles containing luciferase and GFP reporters (Capital Biosciences, Rockville, MD, USA) were infected into shAMPKα knockdown ovarian cancer cells. Cell sorting for high fluorescence was performed on transfectants by using a BD FACSAria I Cell Sorter (Faculty Core Facility, The University Hong Kong).

### Proteomics, lipidomics, and bioinformatics analysis

LC-MS/MS analysis was carried out on an Orbitrap Fusion Lumos mass spectrometer interfaced with Dionex 3000RSLC nanoLC. The high resolution, high mass accuracy MS data obtained were processed using Maxquant version 1.5.3.30, in which MS data analyzed in triplicates for each condition were searched using the Andromeda algorithm against Uniprot Human protein database. Appropriate parameter settings to obtain peptide and protein data using 0.1% FDR at peptide and protein level. Data visualization and statistical data analysis were performed by Perseus software version 1.5.4.1. Differential proteins were subjected to Gene Ontology enrichment analysis using PANTHER^58^, and Ingenuity pathway analysis (IPA) software (Qiagen Bioinformatics). The fatty acid profiling in ovarian cancer cells co-cultured in OCM and DMEM control was performed by using FFA Kit Ultra on an ultra-performance liquid chromatography coupled to tandem-mass spectrometry (UPLC-MS/MS) system (Metabo-Profile, Shanghai, China).

### Western blot analysis

Western blot analysis was performed as previously described^57^. Briefly, the protein lysates were isolated using cell lysis buffer (Cell Signaling Technology), and separated by 10% SDS-PAGE before transferring to polyvinylidene difluoride (PVDF) membranes. The membranes were then pre-blotted with 5% skimmed milk and followed by probing with primary antibodies. The list of primary antibodies used in this study are available in Supplementary Table 2. The goat anti-rabbit or anti-mouse secondary antibody with horseradish peroxidase-conjugated (Amersham, Uppsala, Sweden) were added. Immunodetection was carried out using ECL^TM^ Western Blotting Detection Reagents (Bio-Rad) and visualized by X-ray film.

### Fluorescent staining

Fluorescent staining on the lipid droplet in the ovarian cancer cells were performed using Nile Red (Molecular Probes, Eugene, OR, USA) according to the manufacturer’s protocol^59^. Cells were seeded in 6-well plate in which a sterile glass coverslip had been placed in advance. After 24 h starvation, cells were treated with OCM or DMEM with 1% FBS for 24 h in the same condition as cell culture. Four percent paraformaldehyde (PFA) was used to fix the cell, and followed by the incubation of Nile Red at a concentration of 1 μg/10 mL 150 mM NaCl for 30 min. Then the coverslip was placed on a microslide with a drop of Fluoroshield^TM^ with DAPI (Sigma-Aldrich, St. Louis, MO, USA) to stain cell nucleus. Inner lipid droplet of ovarian cancer cells was stained into red color while nucleus was stained into blue. Cells were captured by Nikon eclipse Ti-S (Nikon, Tokyo, Japan) fluorescent microscope or Zeiss LSM 780 confocal microscope (Carl Zeiss, Germany). To quantify the fluorescent signal of the lipid droplets, three cells per visual field were randomly selected. Measurements of fluorescence intensity were performed by Carl Zeiss ZEN 2.3 Version 13.0.0.0, with normalization of cytosolic intensity. Mean signal intensities were obtained from at least five visual fields.

### XTT cell proliferation, transwell cell migration/invasion assays

Cell viability or cell growth was evaluated by cell proliferation kit (XTT) (Roche Diagnostics, Indianapolis, USA). Briefly, cells were separately seeded in 96-well plates and were treated with OCM added with 1% FBS for different duration of day. To avoid bias, triplicate wells were performed. A mixture of XTT reagent, which contains PBS, XTT labeling reagent, and electron coupling reagent, was incubated with each well for 4 h at 37 °C. Then the absorbance of each well at 492 nm was detected. The relative cell viability was calculated as a fold change relative to the mean of the first day. The capacities of cell migration and invasion of ovarian cancer cell were performed by Transwell cell migration and invasion assay kit (Merck Millipore, New Orleans, LA, USA) according to the manufacturer’s protocol and pictured by microscopy. Relative migratory cell numbers were counted at least from three randomly chosen.

### Three-dimensional (3D) cell culture drug-sensitivity assay

Ovarian cancer cells were suspended in OCM containing 2% Geltrex™ Matrix (Gibco-BRL, Gaithersburg, MD) and seeded in the Nunclon™ Sphera™ 96U-microplates (Thermo Fisher Scientific Inc., Waltham, Massachusetts) with the super low cell attachment surface at around 2000 cells/well density in triplicate. Cells were subsequently allowed to grow for 1 week before treatments with each indicated drug at serial diluted concentrations or in combination for 72 h. After the corresponding drug treatments, viable cells were detected by luminescent cell viability assay using CellTiter-Glo® 3D cell viability assay (Promega Corporation, Madison, WI).

### Ex vivo omental colonization assay

The procedure of this assay was modified from a previous study^5^. Omentum was surgically resected from sacrificed 6–8-week-old SCID female mice and was placed in pre-cooled PBS. Cells were trypsinized and re-suspended in a concentration of 3 × 10^5^/2 mL in DMEM/F12 (Gibco-BRL, Grand Island, NY, USA) with 20% FBS. Omental tissue was cut into even-sized pieces and spread out drily at the bottom of each well of plate. Two milliliters of cell suspension was added to each well and was incubated at 37 °C and 5% CO_2_ for 24 h. Then the omentum was removed out and placed into a 6-well plate, washing with PBS gently to discard the non-attached cells. Each well was cultured with DMEM/F12 with 20% FBS, and the medium was changed for every 3 days. After 14 days of incubation, ZOE^TM^ Fluorescent Imager (Bio-Rad, Hercules, CA, USA) was used to visualize and captured the tumor nodule formation in omentum.

### In vivo intraperitoneal dissemination mouse model

A highly metastatic ovarian cancer cell line ES-2^60^ was selected for labeling by transfecting the LV-CMV-RLuc-IRES-GFP plasmid (consists of both luciferase and GFP reporters) (Capital Biosciences, Inc., Rockville, MD, USA). Then, the eGFP-labeled ES-2 (1 × 10^6^ cells/200 μL) cells were intraperitoneally (i.p.) injected into 5–6-week-old SCID female mice. Tumor dissemination was observed by a laparotomy procedure^61,62^. Metastatic tissues were examined by histological examination using H&E staining. Blood samples were collected from the facial vein via puncture at the rear of the jawbone. All blood tests for renal and liver function analyses were performed by PathLab Medical Laboratories Ltd. (Hong Kong). All animal experiments were approved by the University of Hong Kong Committee on the Use of Live Animals in Teaching and Research (CULATR No. 3968-16).

### Statistical analysis

All statistical analyses were performed by GraphPad Prism 5.0 (San Diego, CA, USA). Data were analyzed by unpaired *t*-test, and one/two-way analysis of variance (ANOVA) followed by Tukey post hoc comparison. All data were obtained from at least three independent experiments, each performed in triplicate, and data are expressed as mean ± S.E.M. and *p* < 0.05 was considered as statistically significant.

### Reporting summary

Further information on research design is available in the Nature Research Reporting Summary linked to this article.

## Supplementary information

Supplementary Information

Reporting Summary

## Data Availability

The proteomic array, lipidomic profiling datasets, and the source data of charts in the current study are available in figshare^63–65^.
