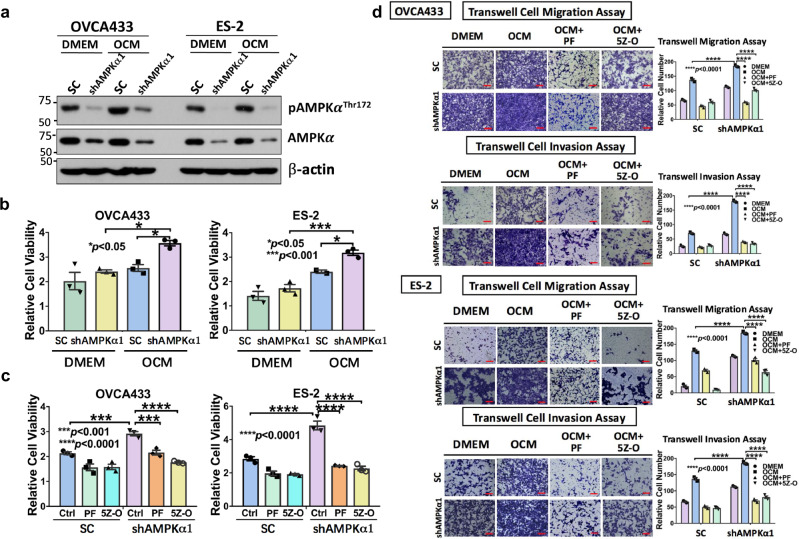# Author Correction: Targeting of lipid metabolism with a metabolic inhibitor cocktail eradicates peritoneal metastases in ovarian cancer cells

**DOI:** 10.1038/s42003-025-07638-3

**Published:** 2025-02-23

**Authors:** Rain R. Chen, Mingo M. H. Yung, Yang Xuan, Shijie Zhan, Leanne L. Leung, Rachel R. Liang, Thomas H. Y. Leung, Huijuan Yang, Dakang Xu, Rakesh Sharma, Karen K. L. Chan, Siew-Fei Ngu, Hextan Y. S. Ngan, David W. Chan

**Affiliations:** 1 The University of Hong Kong Shenzhen Institute of Research and Innovation (HKU-SIRI), Shenzhen, P. R. China; 2 Department of Obstetrics & Gynaecology, LKS Faculty of Medicine, The University of Hong Kong, Hong Kong SAR, P. R. China; 3 Department of Gynecological Oncology, Fudan University Shanghai Cancer Center, Fudan University, Shanghai, 200032 P.R. China; 4 Faculty of Medical Laboratory Science, Ruijin Hospital, School of Medicine, Shanghai Jiao Tong University, Shanghai, 200030 P.R. China; 5 Proteomics & Metabolomics Core Facility, LKS Faculty of Medicine, The University of Hong Kong, Hong Kong SAR, P. R. China

Correction to: *Communications Biology* 10.1038/s42003-019-0508-1, published online 31 July 2019

“The original version of the Article contained an error in Figure 6d as an incorrect image (ES-2 DMEM control in the Transwell Cell Migration Assay) was used. The figure has been corrected.”