# Supplementary material for: Targeting of lipid metabolism with a metabolic inhibitor cocktail eradicates peritoneal metastases in ovarian cancer cells
Source: Commun Biol. 2019 Jul 31;2:281. doi: 10.1038/s42003-019-0508-1 (PMC6668395; doi:10.1038/s42003-019-0508-1)
Supplement: Supplementary file 1 — Supplementary Information [file 42003_2019_508_MOESM1_ESM.pdf]

Supplementary Figure 1

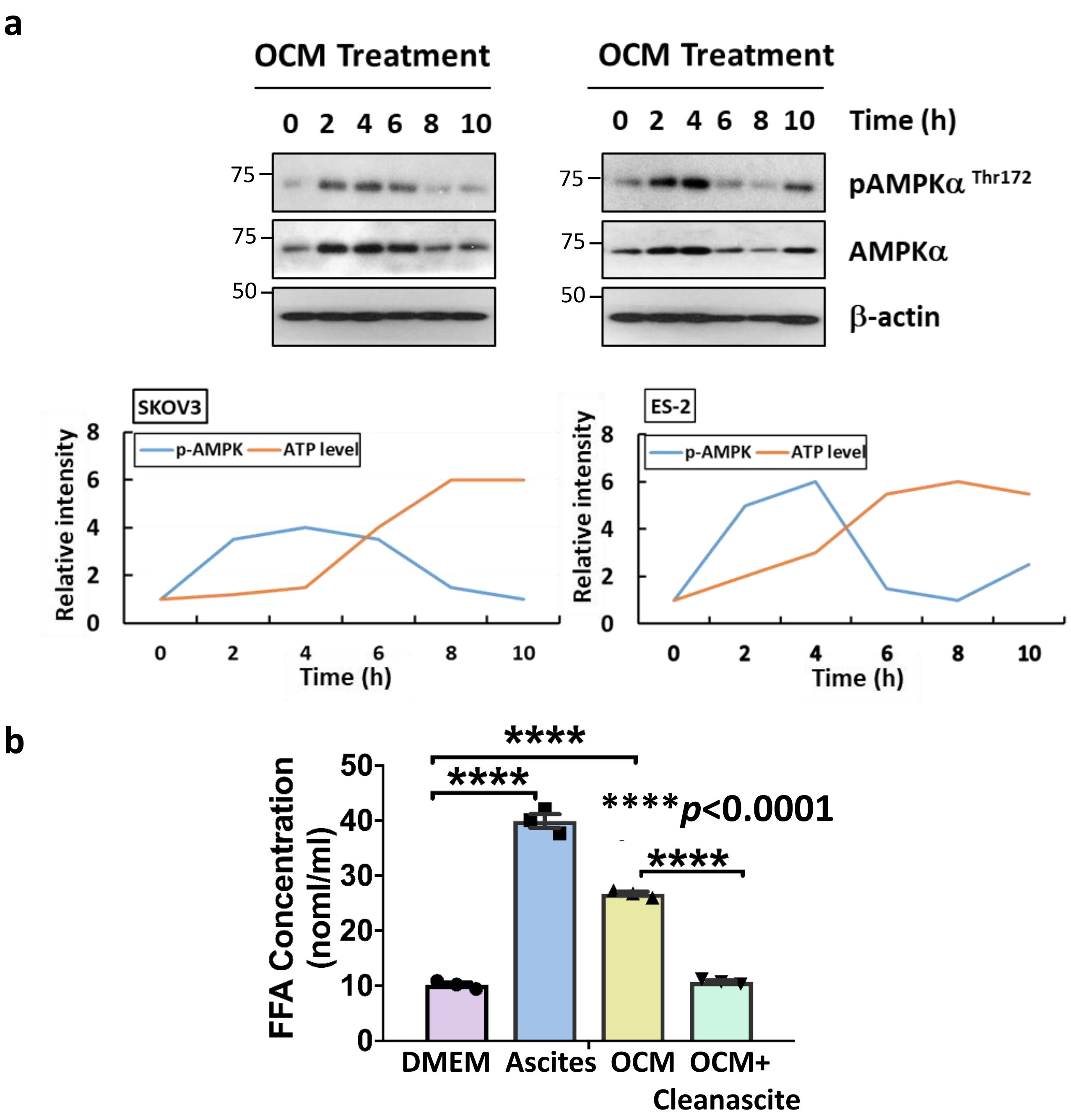

Supplementary Figure 1: (**a**) Western blot analysis revealed that the expression of phospho-AMPK at Thr172 was initially up-regulated when co-cultured with OCM at 0-6 hours in SKOV3 (*left*) and 0-4 hours in ES-2 (*right*), following with a gradual down-regulation up to 10 hours treatment. The expression of AMPK was negatively correlated with the ATP production. The linear graph below showed the reciprocal relationship between the phosphorylation of AMPK and the ATP production. (**b**) The content of free fatty acids (>8-Carbon) in fresh ascites, OCM, OCM with Cleanascites (1:4 mixed with Cleanascite for an hour before centrifugation) were quantified by Free Fatty Acid Assay as compared with DMEM.

Supplementary Figure 2

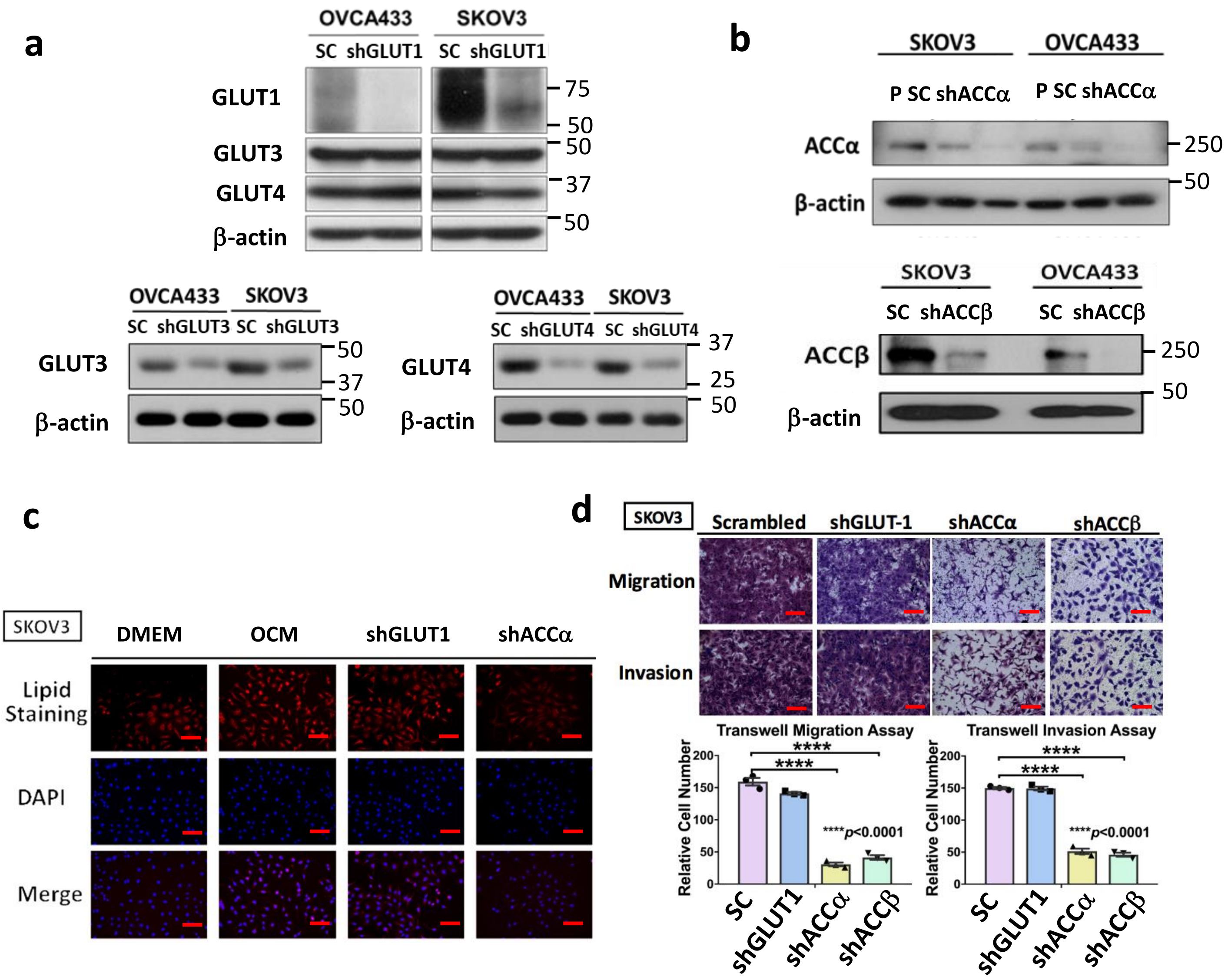

Supplementary Figure 2: (**a**) Western blot analysis indicates that stable knockdown of GLUT1 does not alter the expression of the other 2 dominant Glut isoforms GLUT3 or GLUT4 in SKOV3 or OVCA433 cells (*upper*). Western blot analysis also shows the stable knockdown of GLUT3 and GLUT4 in SKOV3 and OVCA433 (*lower*). (**b**) Western blot analysis shows the stable knockdown of ACCα (*upper*) and ACCβ (*lower*) in OVCA433 and SKOV3. All knockdown were performed by using lentiviral shRNAi particles (Santa Cruz) and the negative controls of each cell lines were established by lentiviral scrambled shRNAi (Santa Cruz). P, parental, SC, scrambled control. (**c**) Immunofluorescent and lipid staining analyses show that the lipid droplet formation in OCM compared with DMEM control in SKOV3 cells with stably knockdown of GLUT1, ACCα, and ACCβ. SC=scrambled control. Scale bar=50 μm. (**d**) Transwell cell migration/invasion assays show that cell migration and invasion rates in SKOV3 cells with stably knockdown of GLUT1, ACCα, and ACCβ. SC=scrambled control. Scale bar=50 μm.

Supplementary Figure 3

a

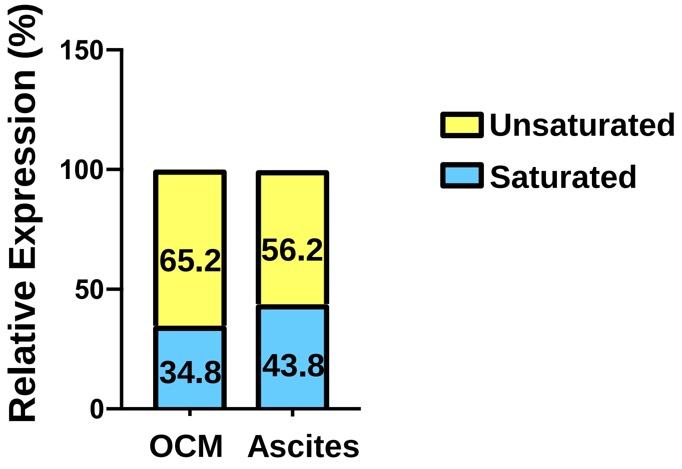

b

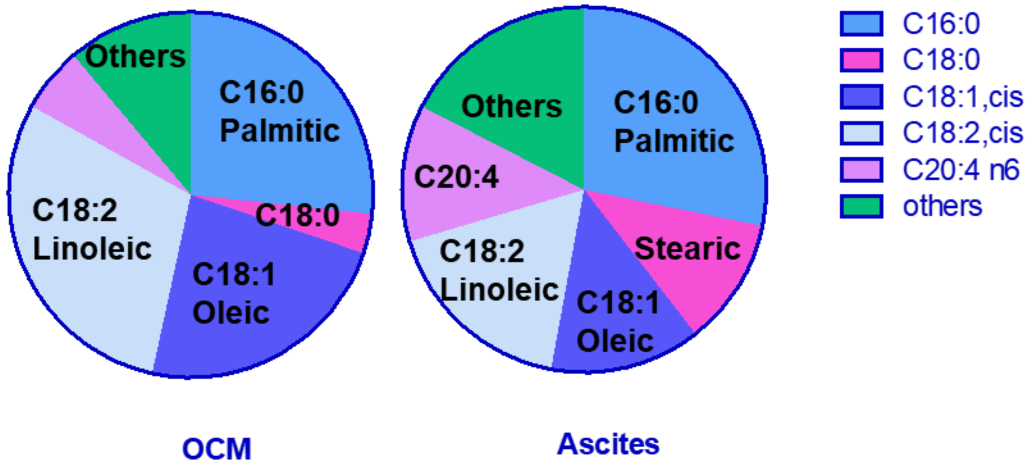

Supplementary Figure 3: The Lipidome analysis of fatty acid compositions performed by Center for Genomic Sciences, LKS Faculty of Medicine, HKU. (**a**) The bar chart shows the amount of unsaturated and saturated fatty acids, and (**b**) The percentage chart shows the amount of major fatty acids found in OCM (N=3) and ascites (N=2).

Supplementary Figure 4

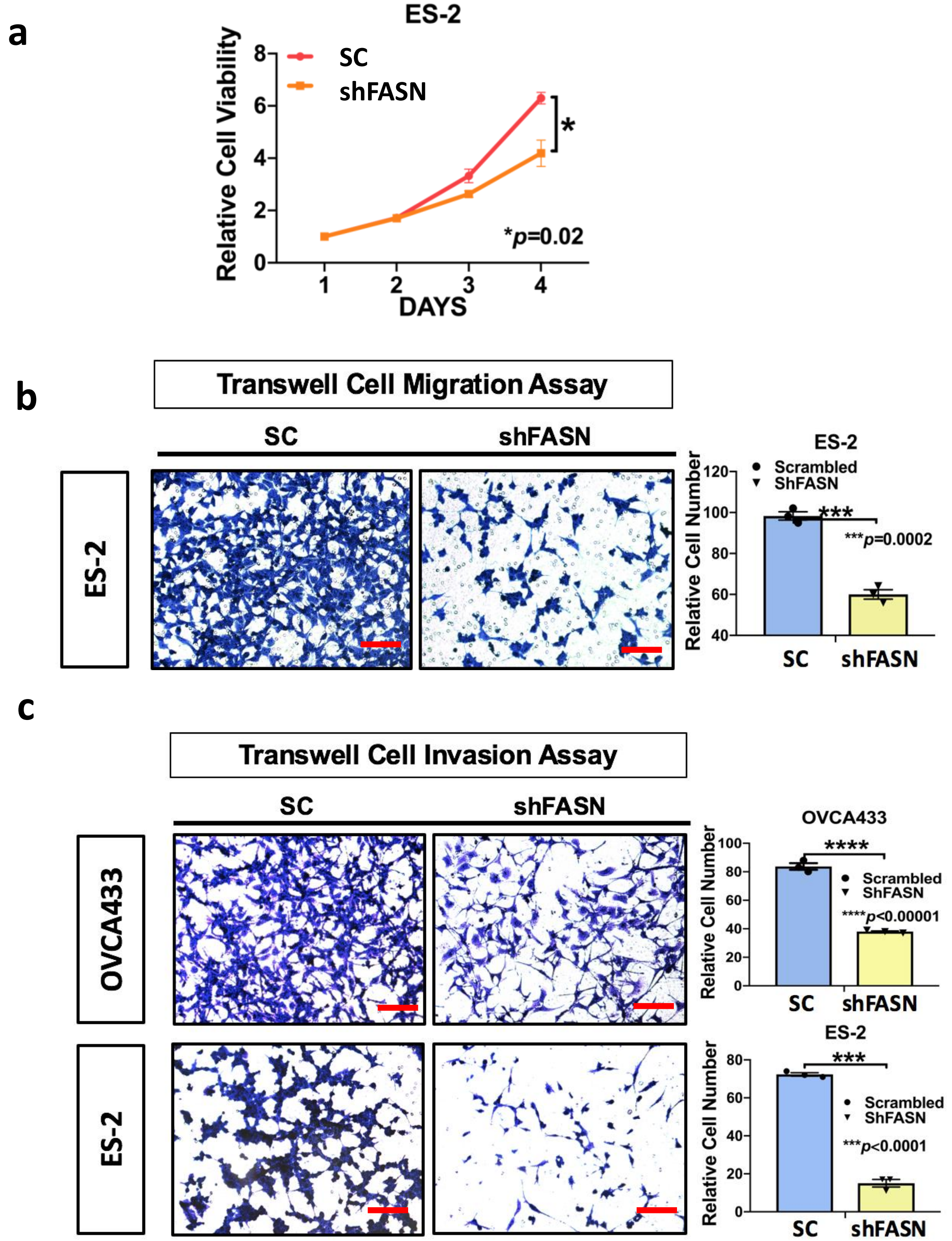

Supplementary Figure 4: The depletion of FASN by shRNAi knockdown reduces the oncogenic properties of ovarian cancer cells in OCM. (a) The XTT cell proliferation assay shows the cell growth rate of ES-2 with or without FASN knockdown co-cultured in OCM for 4 days. (b) Transwell cell migration and (c) transwell cell invasion assays demonstrate that OVCA433 and ES-2 cells with or without FASN knockdown loss both cell migratory and invasive capacities in The stained cells were counted from 4 selected fields randomly. Representative images and quantitative results of cell migration and invasion were shown. SC=scrambled control. Scale bar=50 µm.

Supplementary Figure 5

a

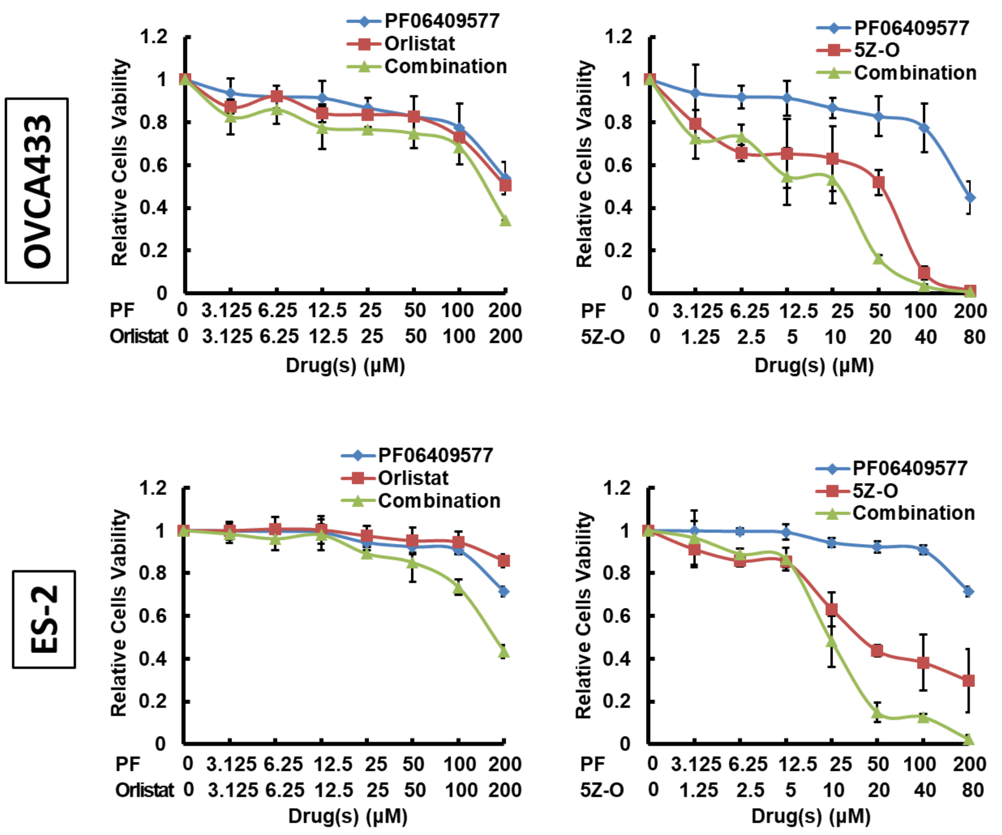

b

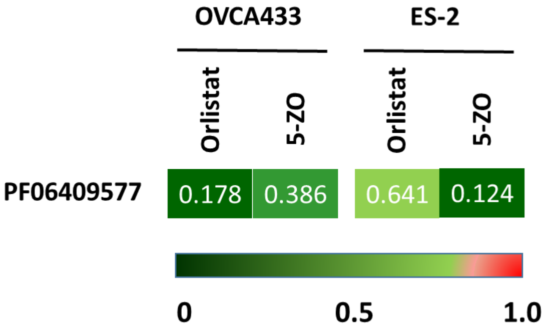

Supplementary Figure 5: The dose response curves show (**a**) OVCA433, and (**b**) ES-2 3D spheroids were cultured in Geltrex™ Matrix with co-treatment of the AMPK activator PF-06409577, the FASN inhibitor Orlistat, and the TAK1 inhibitor (5z)-7-oxozeaenol alone or in combination. The heatmap shows Combination Index (CI) values were calculated by the algorithm derived by Chou and Talalay using the CalcuSyn software (Biosoft, Version 2.1). Combination index (CI) = CI < 1, synergism; CI = 1, additive effect; CI > 1, antagonism.

Supplementary Figure 6

c

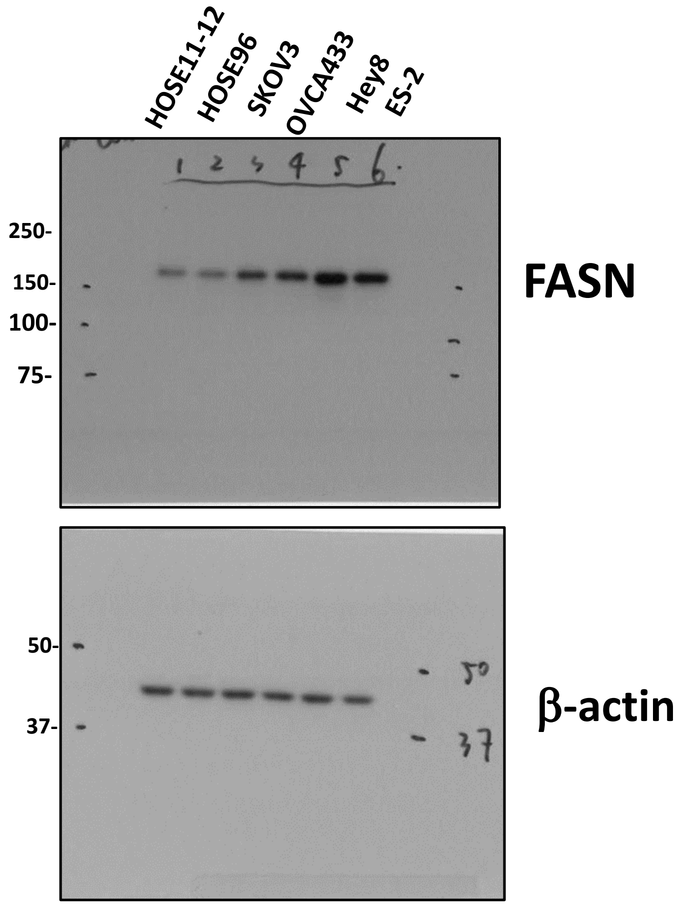

d

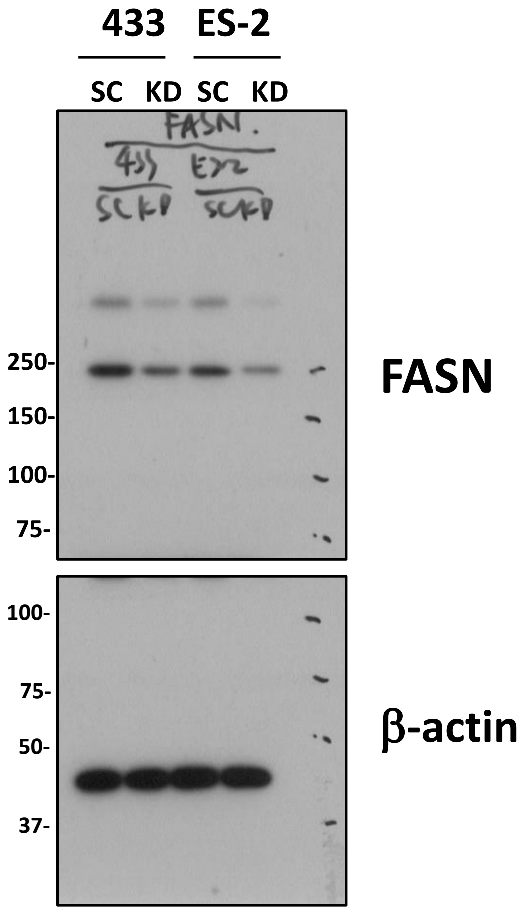

Supplementary Fig. 6: Uncropped western blots of Fig. 4. Precision Plus Protein™ Standards (Bio-Rad) was used for the protein size markers.

Supplementary Figure 7

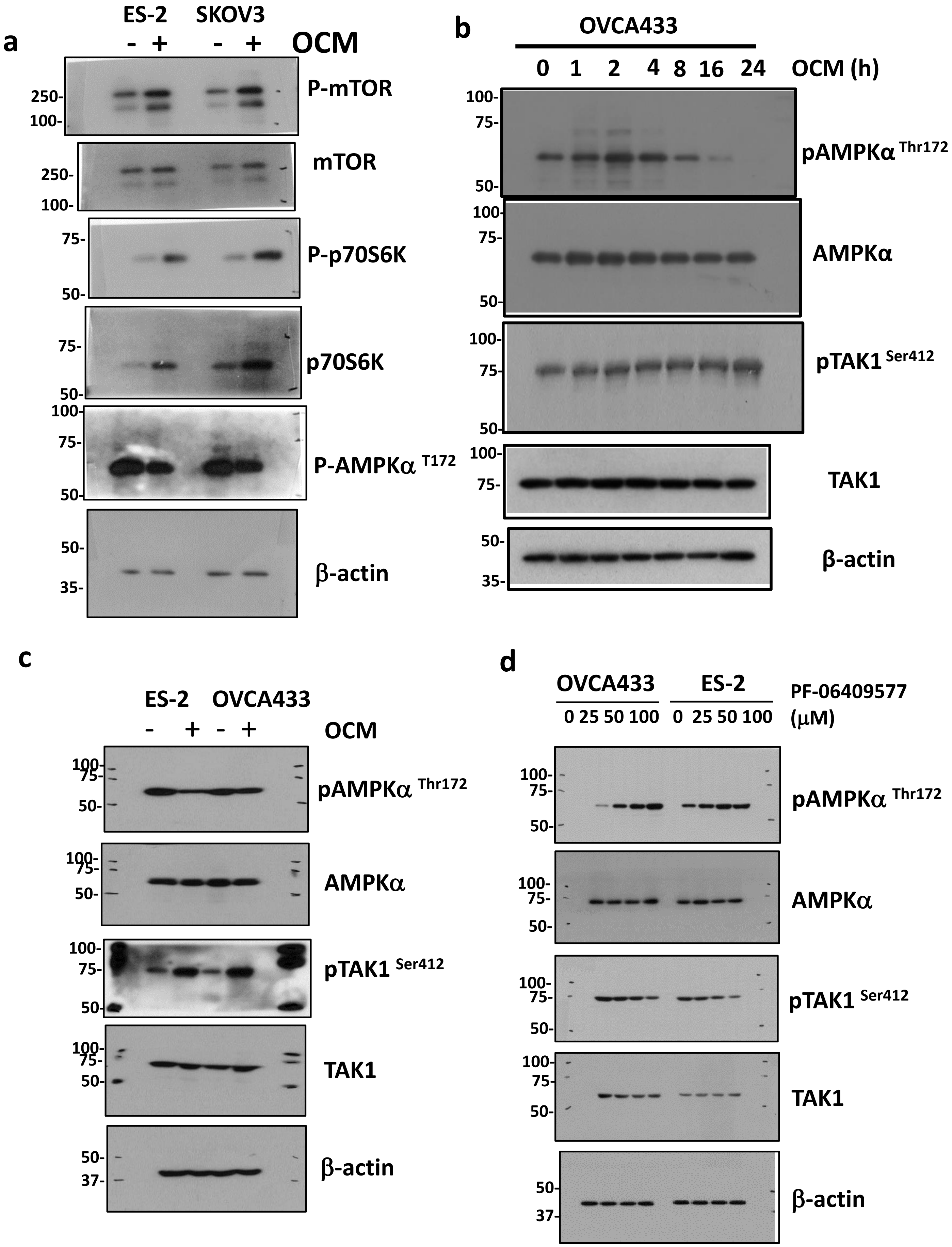

Supplementary Fig. 7: Uncropped western blots of Fig. 5. Precision Plus Protein™ Standards (Bio-Rad) was used for the protein size markers.

Supplementary Figure 7

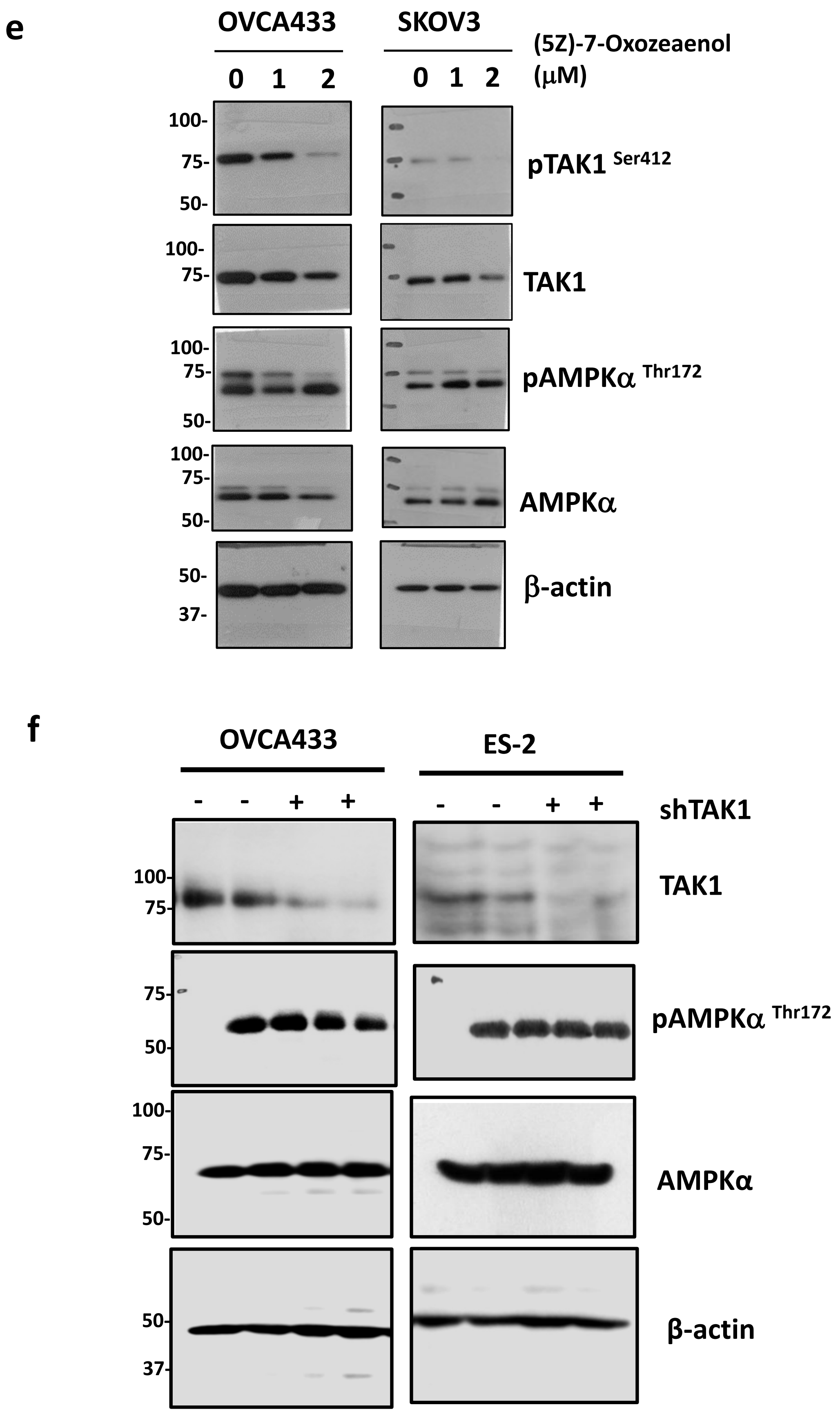

Supplementary Fig. 7: Uncropped western blots of Fig. 5. Precision Plus Protein™ Standards (Bio-Rad) was used for the protein size markers.

Supplementary Figure 8

a

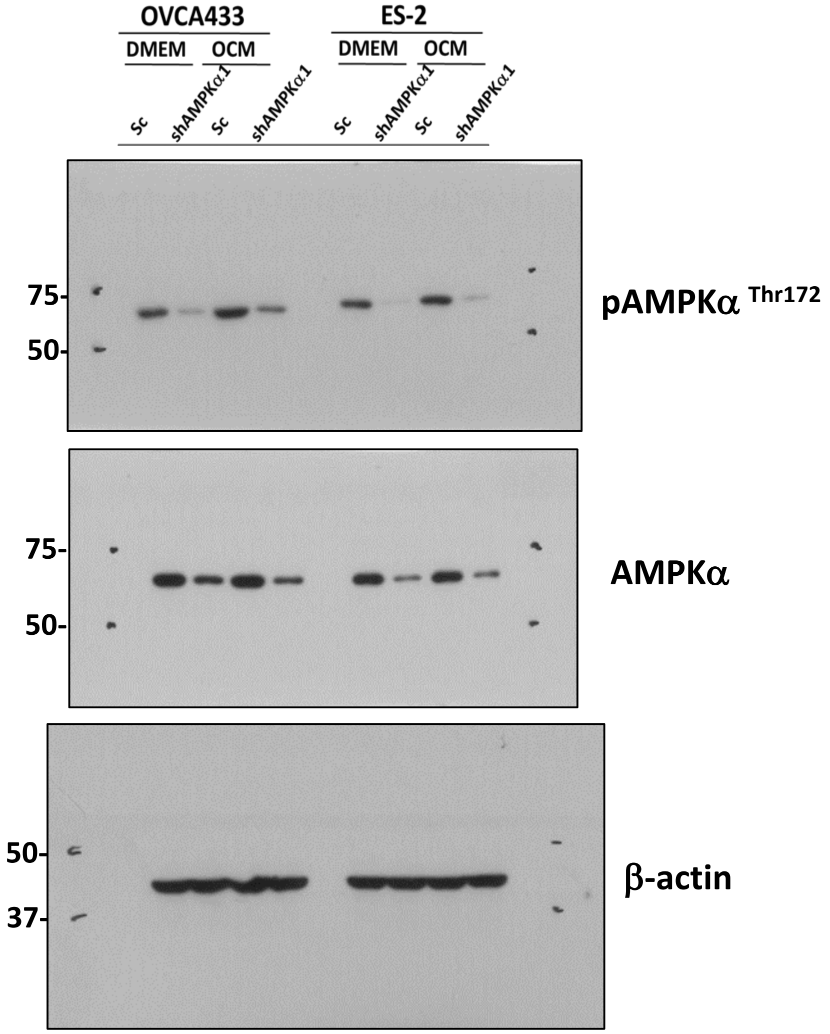

Supplementary Fig. 8: Uncropped western blots of Supplementary Fig. 6. Precision Plus Protein™ Standards (Bio-Rad) was used for the protein size markers.

Supplementary Figure 9

a

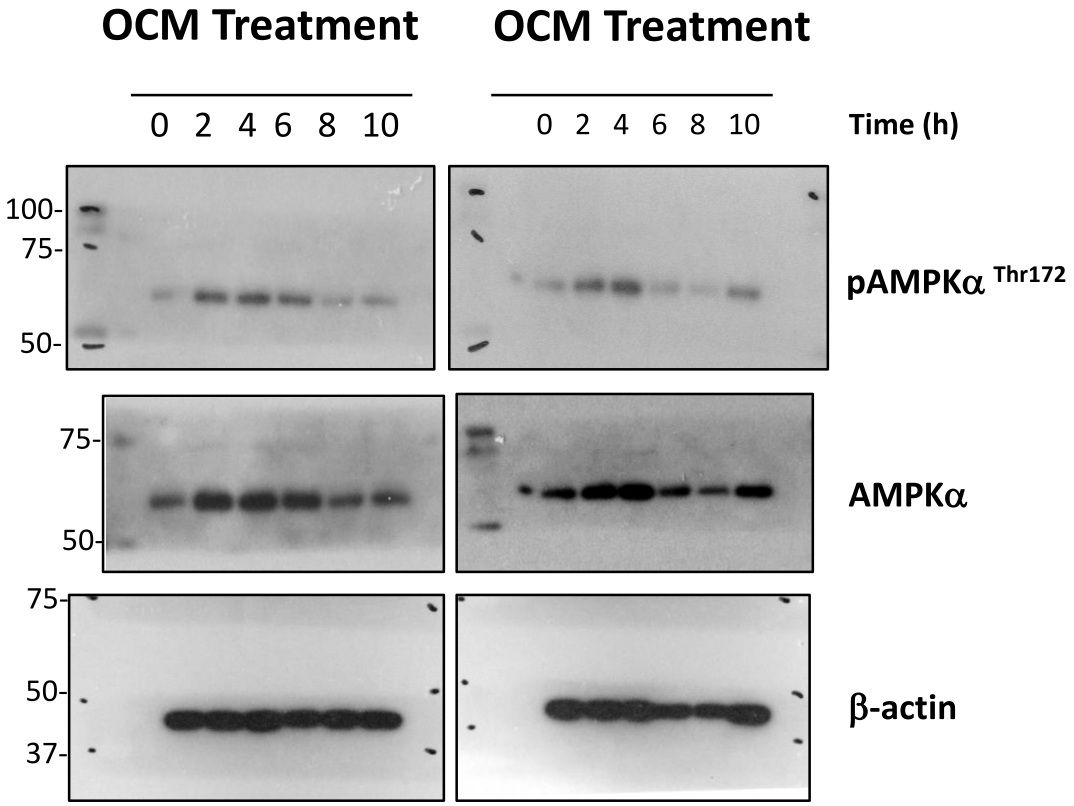

Supplementary Fig. 9: Uncropped western blots of Supplementary Fig. 1. Precision Plus Protein™ Standards (Bio-Rad) was used for the protein size markers.

Supplementary Figure 10

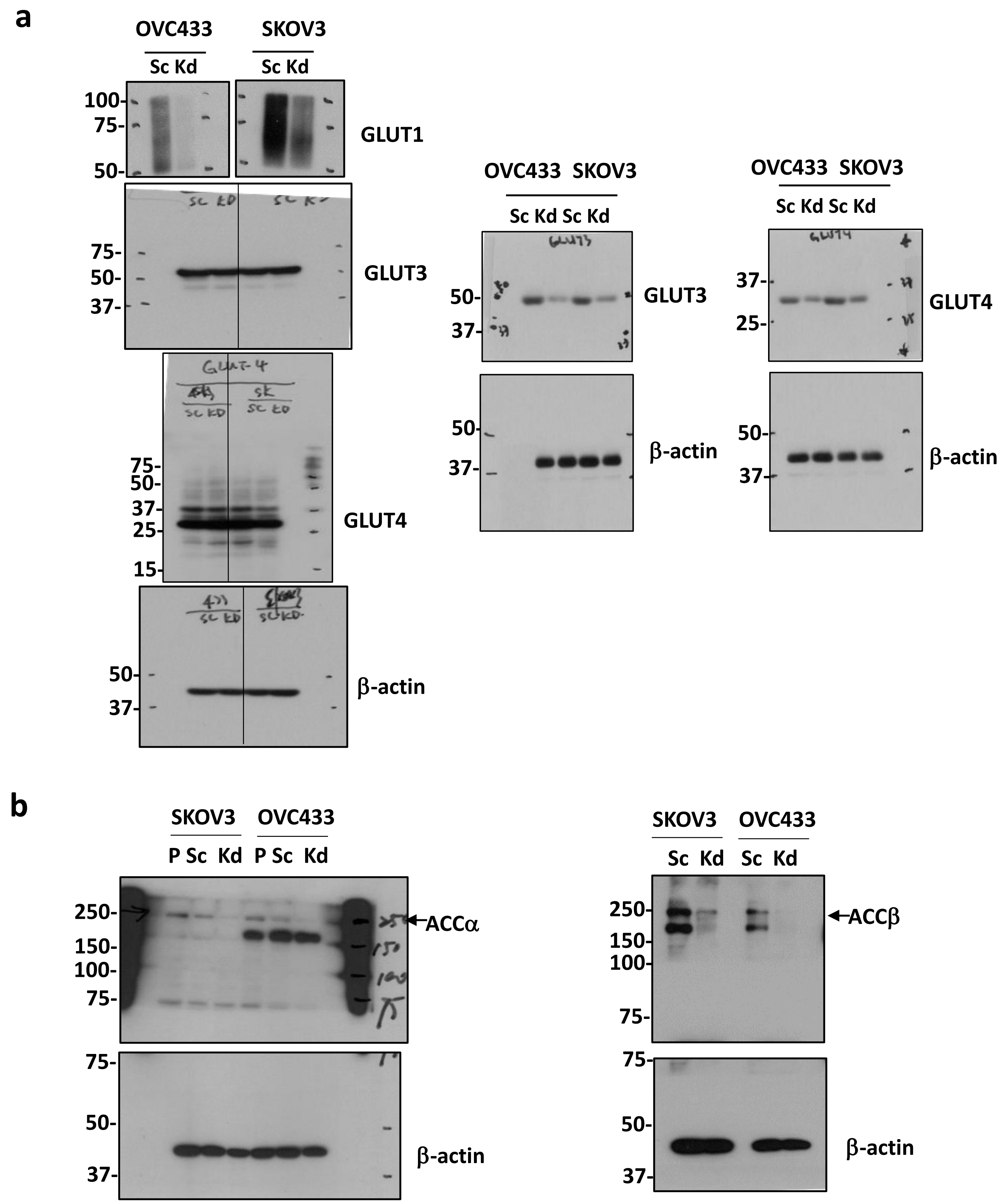

Supplementary Fig. 10: Uncropped western blots of Supplementary Fig. 2. Precision Plus Protein™ Standards (Bio-Rad) was used for the protein size markers.

Supplementary Table 1: Kits/Reagents and drugs used in this study**.**

**Kits/Assays**	**Companies**
Cleanascite™ Lipid Removal Reagent	Biotech Support Group, Monmouth Junction, NJ, USA
The Lipolysis Assay Kit (Colorimetric)	Abcam, Cambridge, MA, USA
Free Fatty Acid Quantification Assay Kit (ab65341)	Abcam, Cambridge, MA, USA
Luminescent ATP Detection Assay Kit (ab113849)	Abcam, Cambridge, MA, USA
Glucose Uptake Assay Kit (Colorimetric) (ab136955)	Abcam, Cambridge, MA, USA
PF-06409577 (Cat. No. 6114)	Tocris Bioscience, Minneapolis, MN, USA
Orlistat (Cat. No. 3540)	Tocris Bioscience, Minneapolis, MN, USA
(5Z)-7-Oxozeaenol (Cat. No. O9890)	Sigma-Aldrich, St. Louis, MO, USA

Supplementary Table 2: Antibodies used for Western blotting**.**

Antibodies	Concentration	Companies
AMPKα	1:1000	Cell Signaling Technology, Danvers, MA, USA
phospho-AMPKα ^{Thr172}	1:1000	Cell Signaling Technology, Danvers, MA, USA
ACCα	1:1000	Cell Signaling Technology, Danvers, MA, USA
ACCβ	1:1000	Cell Signaling Technology, Danvers, MA, USA
phospho-ACC ^{Ser79}	1:1000	Cell Signaling Technology, Danvers, MA, USA
TAK1	1:1000	Cell Signaling Technology, Danvers, MA, USA
phospho-TAK1 ^{Ser412}	1:1000	Cell Signaling Technology, Danvers, MA, USA
phospho-mTOR	1:1000	Cell Signaling Technology, Danvers, MA, USA
mTOR	1:1000	Cell Signaling Technology, Danvers, MA, USA
phospho-pP70S6K	1:1000	Cell Signaling Technology, Danvers, MA, USA
pP70S6K	1:1000	Cell Signaling Technology, Danvers, MA, USA
phospho-IKKα/β $^{Ser176/Ser180}$	1:1000	Cell Signaling Technology, Danvers, MA, USA
FASN	1:1000	Cell Signaling Technology, Danvers, MA, USA
GLUT1	1:1000	Abcam, Cambridge, MA, USA
GLUT3	1:1000	Abcam, Cambridge, MA, USA
GLUT4	1:1000	Abcam, Cambridge, MA, USA
β-actin	1:1000	Sigma-Aldrich, St. Louis, MO, USA
