# Supplementary material for: Targeting of lipid metabolism with a metabolic inhibitor cocktail eradicates peritoneal metastases in ovarian cancer cells
Source: Commun Biol. 2019 Jul 31;2:281. doi: 10.1038/s42003-019-0508-1 (PMC6668395; doi:10.1038/s42003-019-0508-1)
Supplement: Supplementary file 2 — Reporting Summary [file 42003_2019_508_MOESM2_ESM.pdf]

nature research

Corresponding author(s): Dr. David W Chan and Prof. Hextan YS Ngan

Last updated by author(s): Jun 7, 2019

Reporting Summary

Nature Research wishes to improve the reproducibility of the work that we publish. This form provides structure for consistency and transparency in reporting. For further information on Nature Research policies, see Authors & Referees and the Editorial Policy Checklist.

Statistics

For all statistical analyses, confirm that the following items are present in the figure legend, table legend, main text, or Methods section.

n/a	Confirmed	
☐	☒	The exact sample size (*n*) for each experimental group/condition, given as a discrete number and unit of measurement
☐	☒	A statement on whether measurements were taken from distinct samples or whether the same sample was measured repeatedly
☐	☒	The statistical test(s) used AND whether they are one- or two-sided<br>*Only common tests should be described solely by name; describe more complex techniques in the Methods section.*
☒	☐	A description of all covariates tested
☒	☐	A description of any assumptions or corrections, such as tests of normality and adjustment for multiple comparisons
☐	☒	A full description of the statistical parameters including central tendency (e.g. means) or other basic estimates (e.g. regression coefficient) AND variation (e.g. standard deviation) or associated estimates of uncertainty (e.g. confidence intervals)
☐	☒	For null hypothesis testing, the test statistic (e.g. *F*, *t*, *r*) with confidence intervals, effect sizes, degrees of freedom and *P* value noted<br>*Give P values as exact values whenever suitable.*
☒	☐	For Bayesian analysis, information on the choice of priors and Markov chain Monte Carlo settings
☒	☐	For hierarchical and complex designs, identification of the appropriate level for tests and full reporting of outcomes
☒	☐	Estimates of effect sizes (e.g. Cohen's *d*, Pearson's *r*), indicating how they were calculated

Our web collection on statistics for biologists contains articles on many of the points above.

Software and code

Policy information about availability of computer code

Data collection	Uniprot Human protein database, Gene Ontology enrichment analysis using PANTHER
Data analysis	SPSS 14.0 (SPSS), Ingenuity® Pathway Analysis (IPA®) (QIAGEN Bioinformatics), CalcuSyn (2.11), GraphPad Prism 5.0

For manuscripts utilizing custom algorithms or software that are central to the research but not yet described in published literature, software must be made available to editors/reviewers. We strongly encourage code deposition in a community repository (e.g. GitHub). See the Nature Research guidelines for submitting code & software for further information.

Data

Policy information about availability of data

All manuscripts must include a data availability statement. This statement should provide the following information, where applicable:

- Accession codes, unique identifiers, or web links for publicly available datasets
- A list of figures that have associated raw data
- A description of any restrictions on data availability

The proteomic array, lipidomic profiling datasets, and the source data of charts in the current study are available in figshare http://dx.doi.org/10.6084/m9.figshare.7352000, http://dx.doi.org/10.6084/m9.figshare.7351994, and http://dx.doi.org/10.6084/m9.figshare.7351991, or available from the corresponding author on reasonable request.

Field-specific reporting

Please select the one below that is the best fit for your research. If you are not sure, read the appropriate sections before making your selection.

☒ Life sciences ☐ Behavioural & social sciences ☐ Ecological, evolutionary & environmental sciences

For a reference copy of the document with all sections, see nature.com/documents/nr-reporting-summary-flat.pdf

Life sciences study design

All studies must disclose on these points even when the disclosure is negative.

Sample size	All data were obtained by performing at least three independent experiments. The number of mice =5 for each test group. This sample size was calculated based the assumption on the pilot data and other studies to achieve 95% power to detect differences in means between the two groups using type I error of 0.05.
Data exclusions	No data were excluded. It's because all the data were repeated at least three times independently. The means (±S.E.M.) and p-value were used for all graphs presentation.
Replication	All data were repeated at least three times independently and could be reproduced.
Randomization	All gene enforced expressions, the vector control will be used as negative control. For all shRNAi mediated gene knockdown or miRNA enforced expression, the scrambled controls were used as negative controls. For mice injection of drugs, either carrier solutions or saline or PBS will be used as negative controls.
Blinding	The results of immunohistochemical (IHC) were scored blindly by three independent observers.

Reporting for specific materials, systems and methods

We require information from authors about some types of materials, experimental systems and methods used in many studies. Here, indicate whether each material, system or method listed is relevant to your study. If you are not sure if a list item applies to your research, read the appropriate section before selecting a response.

Materials & experimental systems

n/a	Involved in the study	
☐	☒	Antibodies
☐	☒	Eukaryotic cell lines
☐	☐	Palaeontology
☐	☒	Animals and other organisms
☒	☐	Human research participants
☒	☐	Clinical data

Methods

n/a	Involved in the study	
☒	☐	ChIP-seq
☒	☐	Flow cytometry
☒	☐	MRI-based neuroimaging

Antibodies

Antibodies used	Please see Supplementary Table 2.
Validation	each antibodies were verified by either other studies or data sheets of related companies such as Cell Signaling Technology (https://www.cellsignal.com), and Abcam (https://www.abcam.com)

Eukaryotic cell lines

Policy information about cell lines

Cell line source(s)	Prof. Benjamin Tsang (Department of Obstetrics and Gynecology, University of Ottawa) for providing ovarian cancer cell lines; A2780cp SKOV3 and ES-2, Prof. George Tsao (School of Biomedical Sciences, LKS Faculty of Medicine, The University of Hong Kong) for OVCA433, and Prof Alice Wong (School of Biological Sciences, The University of Hong Kong) for Hey8 cells.
Authentication	In-house short tandem repeat (STR) DNA profiling analysis was used to authenticate the above cell lines
Mycoplasma contamination	All cell lines were detected with mycoplasma free.
Commonly misidentified lines (See ICLAC register)	Nil

Palaeontology

Specimen provenance	*Provide provenance information for specimens and describe permits that were obtained for the work (including the name of the issuing authority, the date of issue, and any identifying information).*
Specimen deposition	*Indicate where the specimens have been deposited to permit free access by other researchers.*
Dating methods	*If new dates are provided, describe how they were obtained (e.g. collection, storage, sample pretreatment and measurement), where they were obtained (i.e. lab name), the calibration program and the protocol for quality assurance OR state that no new dates are provided.*

☐ Tick this box to confirm that the raw and calibrated dates are available in the paper or in Supplementary Information.

Animals and other organisms

Policy information about studies involving animals; ARRIVE guidelines recommended for reporting animal research

Laboratory animals	5-week-old female SCID mice
Wild animals	Nil
Field-collected samples	All SCID mice were housed by HKU's animal laboratory unit using the standard housing facilties.
Ethics oversight	All animal experiments were approved by the University of Hong Kong Committee on the Use of Live Animals in Teaching and Research (CULATR No.3968-16).

Note that full information on the approval of the study protocol must also be provided in the manuscript.
